# L-Rhamnosylation of *Listeria monocytogenes* Wall Teichoic Acids Promotes Resistance to Antimicrobial Peptides by Delaying Interaction with the Membrane

**DOI:** 10.1371/journal.ppat.1004919

**Published:** 2015-05-22

**Authors:** Filipe Carvalho, Magda L. Atilano, Rita Pombinho, Gonçalo Covas, Richard L. Gallo, Sérgio R. Filipe, Sandra Sousa, Didier Cabanes

**Affiliations:** 1 Instituto de Investigação e Inovação em Saúde, Universidade do Porto, Porto, Portugal; 2 Group of Molecular Microbiology, Instituto de Biologia Molecular e Celular, Porto, Portugal; 3 Instituto de Ciências Biomédicas Abel Salazar, Universidade do Porto, Porto, Portugal; 4 Laboratory of Bacterial Cell Surfaces and Pathogenesis, Instituto de Tecnologia Química e Biológica, Universidade Nova de Lisboa, Oeiras, Portugal; 5 Division of Dermatology, Department of Medicine, University of California San Diego, San Diego, California, United States of America; University of Michigan Medical School, UNITED STATES

## Abstract

*Listeria monocytogenes* is an opportunistic Gram-positive bacterial pathogen responsible for listeriosis, a human foodborne disease. Its cell wall is densely decorated with wall teichoic acids (WTAs), a class of anionic glycopolymers that play key roles in bacterial physiology, including protection against the activity of antimicrobial peptides (AMPs). In other Gram-positive pathogens, WTA modification by amine-containing groups such as D-alanine was largely correlated with resistance to AMPs. However, in *L*. *monocytogenes*, where WTA modification is achieved solely *via* glycosylation, WTA-associated mechanisms of AMP resistance were unknown. Here, we show that the L-rhamnosylation of *L*. *monocytogenes* WTAs relies not only on the *rmlACBD* locus, which encodes the biosynthetic pathway for L-rhamnose, but also on *rmlT* encoding a putative rhamnosyltransferase. We demonstrate that this WTA tailoring mechanism promotes resistance to AMPs, unveiling a novel link between WTA glycosylation and bacterial resistance to host defense peptides. Using *in vitro* binding assays, fluorescence-based techniques and electron microscopy, we show that the presence of L-rhamnosylated WTAs at the surface of *L*. *monocytogenes* delays the crossing of the cell wall by AMPs and postpones their contact with the listerial membrane. We propose that WTA L-rhamnosylation promotes *L*. *monocytogenes* survival by decreasing the cell wall permeability to AMPs, thus hindering their access and detrimental interaction with the plasma membrane. Strikingly, we reveal a key contribution of WTA L-rhamnosylation for *L*. *monocytogenes* virulence in a mouse model of infection.

## Author Summary

*Listeria monocytogenes* is a foodborne bacterial pathogen that preferentially infects immunocompromised hosts, eliciting a severe and often lethal disease. In humans, clinical manifestations range from asymptomatic intestinal carriage and gastroenteritis to harsher systemic states of the disease such as sepsis, meningitis or encephalitis, and fetal infections. The surface of *L*. *monocytogenes* is decorated with wall teichoic acids (WTAs), a class of carbohydrate-based polymers that contributes to cell surface-related events with implications in physiological processes, such as bacterial division or resistance to antimicrobial peptides (AMPs). The addition of other molecules to the backbone of WTAs modulates their chemical properties and consequently their functionality. In this context, we studied the role of WTA tailoring mechanisms in *L*. *monocytogenes*, whose WTAs are strictly decorated with monosaccharides. For the first time, we link WTA glycosylation with AMP resistance by showing that the decoration of *L*. *monocytogenes* WTAs with l-rhamnose confers resistance to host defense peptides. We suggest that this resistance is based on changes in the permeability of the cell wall that delay its crossing by AMPs and therefore promote the protection of the bacterial membrane integrity. Importantly, we also demonstrate the significance of this WTA modification in *L*. *monocytogenes* virulence.

## Introduction

*Listeria monocytogenes* (*Lm*) is a ubiquitous Gram-positive bacterium and the causative agent of listeriosis, a human foodborne disease with high incidence and morbidity in immunocompromised hosts and other risk groups, such as pregnant women, neonates and the elderly. Clinical manifestations range from febrile gastroenteritis to septicemia, meningitis and encephalitis, as well as fetal infections that can result in abortion or postnatal health complications [1]. The most invasive and severe forms of the disease are a consequence of the ability of this pathogen to overcome important physiological barriers (intestinal epithelium, blood-brain barrier and placenta) by triggering its internalization and promoting its intracellular survival into phagocytic and non-phagocytic cells. Once inside a host cell, a tightly coordinated life cycle, whose progression is mediated by several specialized bacterial factors, enables *Lm* to proliferate and spread to neighboring cells and tissues [2, 3].

The *Lm* cell wall is composed of a thick peptidoglycan multilayer that serves as a scaffold for the anchoring of proteins, among which are several virulence factors [4], and of glycopolymers such as teichoic acids, which account for up to 70% of the protein-free cell wall mass [5, 6]. These anionic polymers are divided into membrane-anchored teichoic acids (lipoteichoic acids, LTAs) and peptidoglycan-attached teichoic acids (wall teichoic acids, WTAs). In *Listeria*, WTAs are mainly composed of repeated ribitol-phosphate subunits, whose hydroxyl groups can be substituted with a diversity of monosaccharides [5]. While the polymer structure and the chemical identity of the substituent groups of LTAs are rather conserved across listeriae [7, 8], they display a high variability in WTAs, even within the same species [9]. Specific WTA substitution patterns are characteristic of particular *Lm* serotypes: *N*-acetylglucosamine is common to serogroups 1/2 and 3, and to serotype 4b, but serogroup 1/2 also contains l-rhamnose, whereas serotype 4b displays d-glucose and d-galactose [10]. The broad structural and chemical similarity of LTAs and WTAs results in a considerable degree of functional redundancy, which has complicated the characterization of these macromolecules and the assignment of specific biological roles. However, studies on Gram-positive bacteria have revealed their contribution to important physiological functions (e.g. cell envelope cationic homeostasis [11], regulation of autolysin activity [12], assembly of cell elongation and division machineries [13], defense against antimicrobial peptides [14]) and to virulence-promoting processes, such as adhesion and colonization of host tissues [15, 16].

Antimicrobial peptides (AMPs) are a large family of small peptides (<10 kDa) produced by all forms of living organisms [17], which constitute a major player of the innate immune response against microbial pathogens. Despite their structural diversity, the majority of AMPs share both cationic and amphipathic properties that favor respectively their interaction with the negatively charged prokaryotic surface and insertion into the plasma membrane [17, 18]. Subsequent pore formation or other AMP-mediated membrane-disrupting mechanisms induce bacterial death through direct cell lysis or deleterious interaction with intracellular targets [19]. Bacteria have evolved multiple strategies to avert killing by AMPs [20, 21]. One strategy consists in the modification of their cell surface charge, a process achieved mainly by masking anionic glycopolymers with positively charged groups, thus decreasing their affinity to AMPs. In Gram-positive pathogens, d-alanylation of teichoic acids is a well-characterized mechanism and was demonstrated to be important for bacterial resistance to host-secreted AMPs [22, 23]. In contrast, the contribution of WTA glycosylation mechanisms in AMP resistance has not yet been investigated.

We have previously reported genome-wide transcriptional changes occurring in *Lm* strain EGD-e during mouse infection [24]. Our analysis revealed an elevated *in vivo* expression of the *lmo1081-1084* genes, here renamed as *rmlACBD* because of the high homology of the corresponding proteins with enzymes of the l-rhamnose biosynthesis pathway. In this work, we show that the decoration of *Lm* WTAs with l-rhamnose requires the expression of not only the *rmlACBD* locus but also of *rmlT*, an upstream-flanking gene encoding a putative rhamnosyltransferase. We also demonstrate that *Lm* becomes more susceptible to AMPs in the absence of WTA l-rhamnosylation and predict that this effect is due to an increase of the *Lm* cell wall permeability to these bactericides, which results in a faster disruption of the plasma membrane integrity with lethal consequences for the bacterial cell. Importantly, we present evidence that this WTA tailoring process is required for full-scale *Lm* virulence in the mouse model of infection.

## Results

### The *rmlACBD* locus is required for the presence of l-rhamnose in *Lm* WTAs

**Fig 1:**
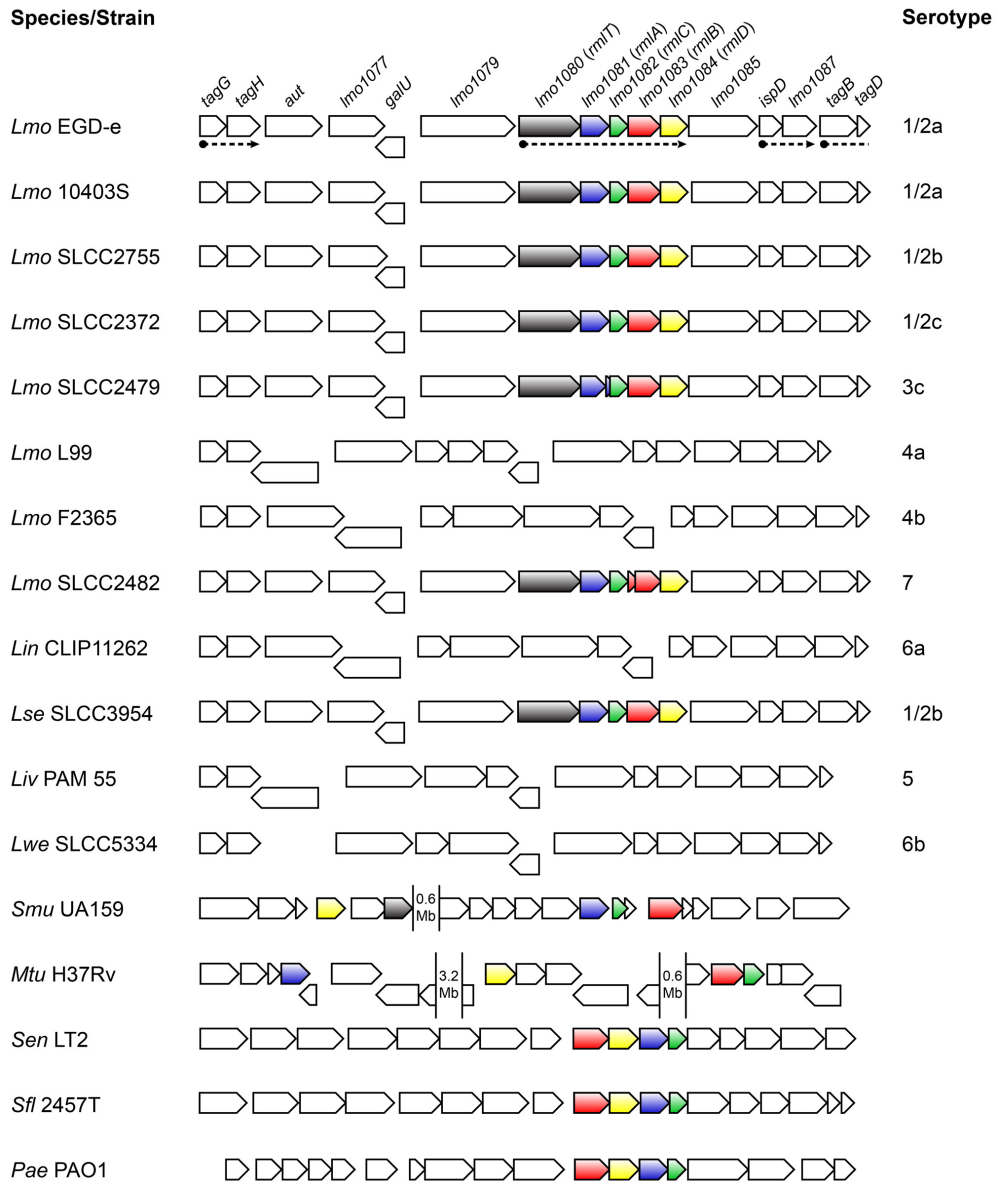
Genes encoding the l-rhamnose biosynthesis pathway are distributed in listeriae and other bacterial species. Comparison of the genomic organization of the l-rhamnose pathway genes in the genus *Listeria* and other bacteria. The corresponding species and strains are indicated on the left (*Lmo*, *Listeria monocytogenes*; *Lin*, *Listeria innocua*; *Lse*, *Listeria seeligeri*; *Liv*, *Listeria ivanovii*; *Lwe*, *Listeria welshimeri*; *Smu*, *Streptococcus mutans*; *Mtu*, *Mycobacterium tuberculosis*; *Sen*, *Salmonella enterica* serovar Typhimurium; *Sfl*, *Shigella flexneri*; *Pae*, *Pseudomonas aeruginosa*) and listerial serotypes are indicated on the right. Genes are represented by boxed arrows and their names are provided for strain EGD-e. Operons are underlined by dashed arrows and homologs of the *rml* genes are shown with identical colors. Numbered gaps indicate the genetic distance (Mb, mega base pairs) between *rml* genes located far apart in the chromosome. Bacterial genomic sequences were obtained from NCBI database and chromosomal alignments assembled using Microbial Genomic context Viewer and Adobe Illustrator.

To identify new *Lm* genes potentially critical for the infectious process, we previously performed the first *in vivo* transcriptional profiling of *Lm* EGD-e. Among the *Lm* genes displaying the largest increase in transcription throughout infection, we identified a set of previously uncharacterized genes that are included in a pentacistronic operon (*lmo1080* to *lmo1084*) [25]. This operon is found in *L*. *monocytogenes* strains belonging to serogroups 1/2, 3 and 7, and is absent from serogroup 4 strains [26] (Fig 1). Interestingly, aside from *Listeria seeligeri* 1/2b strains, this locus is not found in any other *Listeria* spp., such as the nonpathogenic *Listeria innocua* or the ruminant pathogen *Listeria ivanovii*, which pinpoints it as a genetic feature of a particular subset of pathogenic *Listeria* strains and suggests that its expression may be important to *Listeria* pathogenesis in humans.

The four proteins encoded by the *lmo1081-lmo1084* genes share a high amino acid sequence homology with the products of the *rmlABCD* gene cluster. These genes are widely distributed among Gram-negative (e.g. *Salmonella enterica* [27], *Shigella flexneri* [28], *Vibrio cholerae* [29], *Pseudomonas aeruginosa* [30]) and Gram-positive species (e.g. *Mycobacterium tuberculosis* [31], *Streptococcus mutans* [32], *Geobacillus tepidamans* [33], *Lactobacillus rhamnosus* [34]) (Fig 1), the majority of which being known pathogens or potentially pathogenic. Despite the inter-species variability observed in the genetic organization of the *rml* genes, the respective proteins exhibit a remarkable degree of conservation (S1 Table in S1 Text). In light of this, we renamed the *lmo1081*-*lmo1084* genes to *rmlACBD*, respectively (Fig 1).

**Fig 2:**
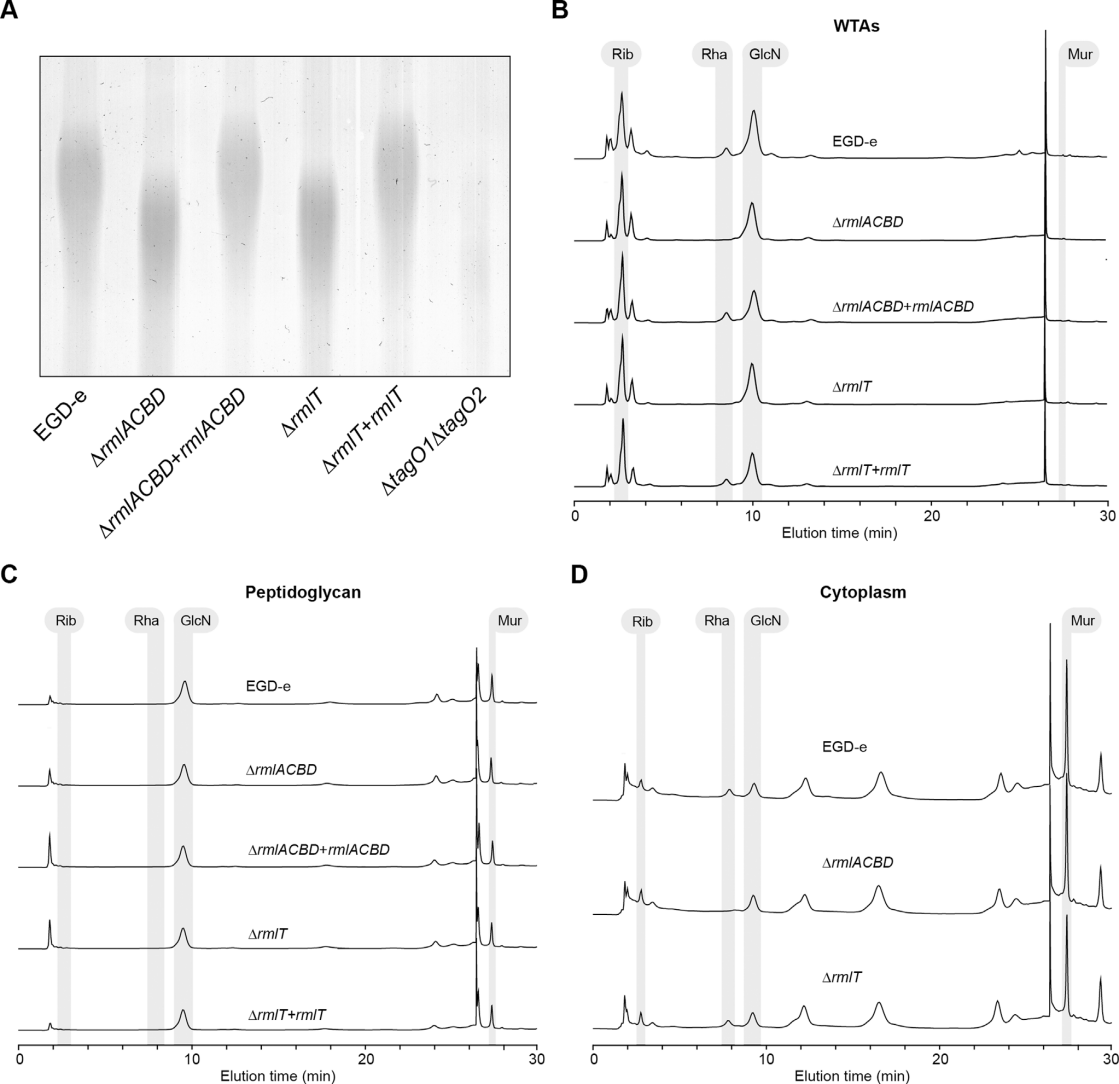
A functional *rml* operon is required for glycosylation of *Lm* WTAs with l-rhamnose. (**A**) Alcian blue-stained 20% polyacrylamide gel containing WTA extracts from logarithmic-phase cultures of different *Lm* strains. (**B–D**) HPAEC-PAD analyses of the sugar composition of the (B) WTA, (C) peptidoglycan and (D) cytoplasmic fractions isolated from the indicated *Lm* strains. Samples were hydrolyzed in 3 M HCl (2 h, 95°C), diluted with water and lyophilized before injection into the HPLC equipment. Standards for ribitol (Rib), l-rhamnose (Rha), glucosamine (GlcN), and muramic acid (Mur) were eluted under identical conditions to allow peak identification.

The RmlABCD proteins catalyze the conversion of glucose-1-phosphate to a thymidine-diphosphate (dTDP)-linked form of l-rhamnose [35] (S1A Fig in S1 Text), which is a component of the WTAs from most *Listeria* strains possessing the *rml* genes [6]. To address the role of *rmlACBD* in *Lm* WTA glycosylation with l-rhamnose, we constructed an *Lm* EGD-e derivative mutant strain lacking the *rmlACBD* locus (Δ*rmlACBD*) (S2A Fig in S1 Text) and investigated if the absence of these genes could affect the WTA l-rhamnosylation status. We prepared WTA hydrolysates from exponential phase cultures of wild type (EGD-e), Δ*rmlACBD* and a complemented Δ*rmlACBD* strain expressing *rmlACBD* from its native promoter within an integrative plasmid (Δ*rmlACBD*+*rmlACBD*). Samples were resolved by native PAGE and the gel stained with Alcian blue to visualize WTA polymer species. A mutant strain unable to synthesize WTAs (Δ*tagO1*Δ*tagO2*) [36] was used to confirm that the detected signal corresponds to WTAs. Compared to the wild type sample, the Δ*rmlACBD* WTAs displayed a shift in migration, which was reverted to a wild type-like profile in WTAs from the Δ*rmlACBD*+*rmlACBD* sample (Fig 2A), indicating that the native WTA composition requires the presence of the *rmlACBD* genes. To confirm this, we investigated the WTA carbohydrate composition from these strains. WTA polymers were isolated from cell walls purified from bacteria in exponential growth phase, hydrolyzed and analyzed by high-performance anion exchange chromatography coupled with pulsed amperometric detection (HPAEC-PAD) to detect monosaccharide species. WTA extracts obtained from Δ*rmlACBD* bacteria completely lacked l-rhamnose, in contrast to those isolated from the parental wild type strain (Fig 2B). The role of *rmlACBD* in *Lm* WTA l-rhamnosylation was definitely confirmed by the analysis of WTAs from Δ*rmlACBD*+*rmlACBD* bacteria, in which l-rhamnose was detected at levels similar to those observed in the wild type sample (Fig 2B). Similar observations were made with purified cell wall samples that contain WTAs still attached to the peptidoglycan matrix (S3A Fig in S1 Text). The absence of muramic acid, one of the peptidoglycan building blocks, from WTA extracts (Fig 2B) indicates that l-rhamnose is specifically associated with WTAs and is not a putative peptidoglycan contaminant. This is corroborated by the absence of l-rhamnose in purified peptidoglycan samples (Fig 2C).

WTAs have been identified as important regulators of peptidoglycan cross-linking and maturation [37]. To investigate if l-rhamnose decoration of WTAs has any involvement in the maturation of the *Lm* peptidoglycan, we performed HPLC analysis of the muropeptide composition of mutanolysin-digested peptidoglycan samples from wild type, Δ*rmlACBD* and Δ*rmlACBD*+*rmlACBD* bacteria. No differences in the nature and relative amount of muropeptide species were observed between strains (S3B Fig in S1 Text), ruling out a role for WTA l-rhamnosylation in the consolidation of the peptidoglycan architecture. Overall, these results confirm that a functional *rmlACBD* locus is required for the association of l-rhamnose with *Lm* WTAs, likely by providing the molecular machinery responsible for the synthesis of l-rhamnose.

### RmlT is required for the incorporation of l-rhamnose into *Lm* WTAs

The *rml* operon in *Lm* includes a fifth gene, *lmo1080*, located upstream of *rmlA* (Fig 1), which codes for a protein similar to the *B*. *subtilis* minor teichoic acid biosynthesis protein GgaB, shown to possess sugar transferase activity [38]. Conserved domain analysis of the translated Lmo1080 amino acid sequence revealed that its N-terminal region is highly similar (e-value 10^–22^) to a GT-A family glycosyltransferase domain (S1B Fig in S1 Text). In GT-A enzymes, this domain forms a pocket that accommodates the nucleotide donor substrate for the glycosyl transfer reaction, and contains a signature DxD motif necessary to coordinate a catalytic divalent cation [39]. This motif is also found within the predicted glycosyltransferase domain sequence of Lmo1080 as a DHD tripeptide (S1B Fig in S1 Text). For these reasons, we investigated whether Lmo1080, which we renamed here RmlT (for l-rhamnose transferase), was involved in the l-rhamnosylation of *Lm* WTAs. We constructed an *Lm* EGD-e mutant strain lacking *rmlT* (S2A Fig in S1 Text) and analyzed the structure and sugar composition of its WTAs as described above. WTAs isolated from Δ*rmlT* bacteria displayed a faster migration in gel (Fig 2A) and did not contain any trace of l-rhamnose (Fig 2B), fully recapitulating the Δ*rmlACBD* phenotype. Reintroduction of a wild type copy of *rmlT* into the mutant strain (Δ*rmlT*+*rmlT*) resulted in a phenotype that resembles that of the wild type strain, with regards to WTA gel migration profile (Fig 2A) and presence of l-rhamnose in the WTA fraction (Fig 2B).

To discard the possibility that the deletion of *rmlT* exerted a negative polar effect on the downstream expression of *rmlACBD*, potentially disrupting the synthesis of l-rhamnose used for WTA glycosylation, we compared the transcription of the *rmlACBD* genes in the wild type and Δ*rmlT Lm* strains by quantitative real-time PCR. Transcript levels were unchanged in the Δ*rmlT* background as compared to the wild type strain (S2B Fig in S1 Text), indicating that the deletion of *rmlT* did not interfere with the transcription of *rmlACBD*. To definitely confirm that *Lm* Δ*rmlT* still holds the capacity to synthesize l-rhamnose, being only incapable to incorporate it in nascent WTA polymers, we evaluated the presence of l-rhamnose in the cytoplasmic compartment of this strain. The intracellular content of early exponential-phase bacteria from the wild type, Δ*rmlACBD* and Δ*rmlT* strains was extracted, hydrolyzed and analyzed by HPAEC-PAD to compare the sugar composition of cytoplasmic extracts. As shown in Fig 2D, a peak corresponding to l-rhamnose was detected in the cytoplasmic samples from the wild type and Δ*rmlT* strains, but not from the Δ*rmlACBD* strain, clearly demonstrating that, as opposed to Δ*rmlACBD* bacteria, Δ*rmlT* bacteria retain a functional l-rhamnose biosynthesis pathway. These results indicate that the depletion of l-rhamnose observed in Δ*rmlT* WTAs is a consequence of the absence of the WTA l-rhamnosyltransferase activity performed by RmlT. Therefore, we propose RmlT as the glycosyltransferase in charge of decorating *Lm* WTAs with l-rhamnose.

### WTA l-rhamnosylation promotes *Lm* resistance to AMPs

**Fig 3:**
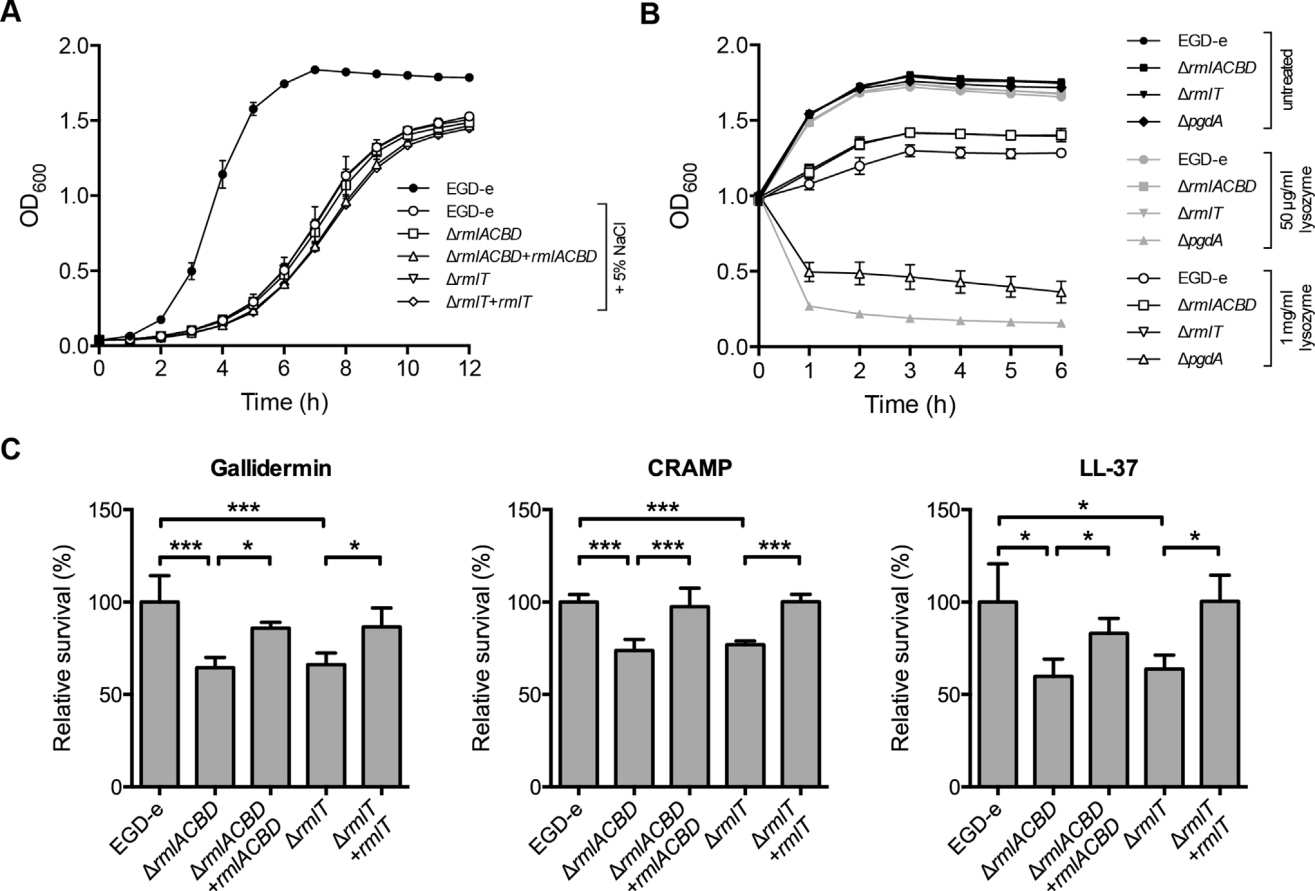
WTA l-rhamnosylation promotes *Lm* resistance against AMPs. (**A**) Growth of *Lm* strains in BHI broth supplemented with 5% NaCl. A growth curve of wild type EGD-e in the absence of 5% NaCl was included as a control for optimal growth. (**B**) Growth of mid-exponential-phase *Lm* strains untreated (black symbols) or challenged with 50 μg/ml (gray symbols) or 1 mg/ml (white symbols) of lysozyme. Optical density of the shaking cultures was monitored spectrophotometrically at 600 nm. (**C**) Quantification of viable bacteria after treatment of mid-exponential-phase *Lm* strains (2 h, 37°C) with gallidermin (1 μg/ml), CRAMP or LL-37 (5 μg/ml). Averaged replicate values from AMP-treated samples were normalized to untreated control samples and the transformed data expressed as the percentage of surviving bacteria relative to wild type *Lm* (set at 100). Data represent mean±SD of three independent experiments. *, *p*≤0.05; ***, *p*≤0.001.

WTAs were previously associated with bacterial resistance against salt stress [40] and host defense effectors, such as lysozyme [37, 41]. We thus investigated the potential involvement of WTA l-rhamnosylation in these processes by assessing the growth of the Δ*rmlACBD* and Δ*rmlT* strains in the presence of high concentrations of either NaCl or lysozyme. As shown in Fig 3A, no significant difference was observed between the growth of the wild type and the two mutant strains in BHI broth containing 5% NaCl. Similarly, no difference was detected between the growth behavior of these strains after the addition of different concentrations of lysozyme (50 μg/ml and 1 mg/ml) to bacterial cultures in the exponential phase (Fig 3B). As expected, we observed an immediate and significant decrease in the survival of the lysozyme-hypersensitive Δ*pgdA* mutant [42] (Fig 3B). These data demonstrate that *Lm* does not require l-rhamnosylated WTAs to grow under conditions of high osmolarity nor to resist the cell wall-degrading activity of lysozyme.

WTAs were also found to be involved in bacterial resistance to host-secreted defense peptides [14, 43]. To investigate the role of WTA l-rhamnosylation in *Lm* resistance to AMPs, we evaluated the *in vitro* survival of wild type, Δ*rmlACBD* and Δ*rmlT Lm*, as well as of the respective complemented strains, in the presence of biologically active synthetic forms of AMPs produced by distinct organisms: gallidermin, a bacteriocin from the Gram-positive bacterium *Staphylococcus gallinarum* [44]; CRAMP, a mouse cathelicidin [45], or its human homolog LL-37 [46]. After two hours of co-incubation with different AMP concentrations, surviving bacteria were enumerated by plating in solid media. The overall survival levels of *Lm* varied with each AMP, evidencing their distinct antimicrobial effectiveness (S4 Fig in S1 Text). However, when compared to the wild type strain, the Δ*rmlACBD* and Δ*rmlT* mutants displayed a consistent decrease in their survival levels in the presence of any of the three AMPs (Fig 3C), in a dose-dependent manner (S4 Fig in S1 Text). Restoring WTA l-rhamnosylation through genetic complementation of the mutant strains resulted in an increase of the survival rate to wild type levels. This result demonstrated the important contribution of l-rhamnosylated WTAs towards *Lm* resistance against AMPs, pointing to a role for WTA glycosylation in bacterial immune evasion mechanisms.

### WTA l-rhamnosylation interferes with *Lm* cell wall crossing by AMPs

**Fig 4:**
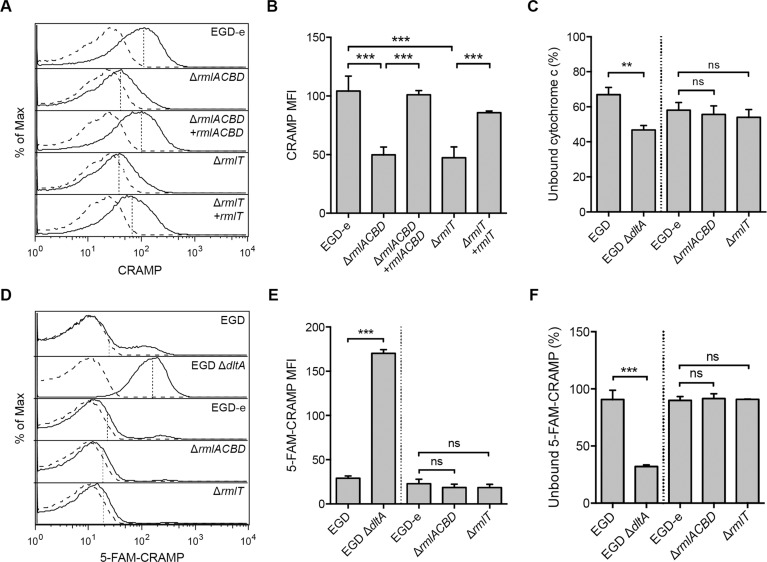
WTA l-rhamnosylation interferes with the *Lm* cell wall crossing by AMPs. (**A and B**) Flow cytometry analysis of *Lm* surface-exposed CRAMP levels in mid-exponential-phase *Lm* strains, following incubation (5 min) in a 5-μg/ml solution of the peptide and immunolabeling with anti-CRAMP and Alexa Fluor 488-conjugated antibodies. (A) Representative experiment showing overlaid histograms of CRAMP-treated (solid line) and untreated (dashed line) samples, with mean fluorescence intensity (MFI) values from treated samples indicated by vertical dashed lines. (B) Mean±SD of the MFI values of CRAMP-treated samples from three independent experiments. (**C**) Cell surface charge analysis of *Lm* strains deficient for WTA l-rhamnosylation as determined by cytochrome c binding assays. Mid-exponential-phase bacteria were incubated with equine cytochrome c (0.5 mg/ml), centrifuged and the supernatant was recovered for spectrophotometric quantification of the unbound protein fraction. Values from *Lm*-containing samples are expressed as the percentage of unbound cytochrome c relative to control samples lacking bacteria. Data represent the mean±SD of three independent experiments. (**D and E**) Flow cytometry analysis of total *Lm*-associated CRAMP levels in mid-exponential-phase *Lm* strains, following incubation (5 min) with a 5-μg/ml solution of fluorescently labeled peptide (5-FAM-CRAMP). (D) Representative experiment showing overlaid histograms of FAM-CRAMP-treated (solid line) and untreated (dashed line) samples, with MFI values from treated samples indicated by vertical dashed lines. (E) Mean±SD of the MFI values of 5-FAM-CRAMP-treated samples from three independent experiments. (**F**) Fluorometric quantification of the unbound CRAMP fraction in the supernatant of suspensions of mid-exponential-phase *Lm* strains, following incubation (5 min) with a 5-μg/ml solution of 5-FAM-CRAMP. Data are expressed as the percentage of unbound fluorescent peptide relative to control samples lacking bacteria, and represent the mean±SD of three independent experiments performed in triplicates. ns = not significant, *p*>0.05; **, *p*≤0.01; ***, *p*≤0.001.

The increased AMP susceptibility of *Lm* strains defective in WTA l-rhamnosylation suggests that this process is required to hinder the bactericidal activity of AMPs. Since AMPs generally induce bacterial death by disrupting the integrity of the plasma membrane, we hypothesized that the higher susceptibility of the Δ*rmlACBD* and Δ*rmlT* mutant strains resulted from an increased AMP-mediated destabilization of the *Lm* membrane. In this context, two scenarios were envisioned: i) AMPs could be binding with higher affinity to the l-rhamnose-deficient *Lm* cell wall, or ii) they could be crossing it at a faster pace, thus reaching the membrane more quickly than in wild type *Lm*. To explore these possibilities, we first investigated the binding affinity of the mouse cathelicidin CRAMP towards *Lm* cell walls depleted of l-rhamnose. For this, we incubated the different *Lm* strains with CRAMP for a short period and analyzed by flow cytometry the amount of *Lm*-bound peptide exposed at the cell surface and accessible for antibody recognition. We detected fluorescence associated with surface-exposed CRAMP in all strains (Fig 4A). However, the mean fluorescence intensity (MFI) values were significantly reduced in both Δ*rmlACBD* and Δ*rmlT* mutants, in comparison to wild type *Lm* and the complemented strains (Fig 4A and 4B). This suggests that CRAMP was less accessible to immunolabeling at the cell surface of *Lm* lacking l-rhamnosylated WTAs.

The affinity of AMPs towards the bacterial surface is driven by electrostatic forces between positively charged peptides and the anionic cell envelope [23]. To determine if variations of the *Lm* surface charge contributed to the reduced amount of CRAMP exposed at the surface of Δ*rmlACBD* and Δ*rmlT* bacteria, we compared the surface charge of *Lm* with or without l-rhamnosylated WTAs. For this, we analyzed the binding of cytochrome c, a small protein with positive charge at physiological conditions (isoelectric point ~10), to the wild type and mutant *Lm* strains. As positive control, we used a mutant strain that cannot modify its LTAs with d-alanine (Δ*dltA*) and, as a result, displays a higher surface electronegativity and a concomitant higher affinity for positively charged compounds [14, 47]. As expected, the level of cytochrome c binding was higher with the Δ*dltA* strain than with the respective wild type strain, as illustrated by a decreased percentage of unbound cytochrome c (Fig 4C). However, no significant difference in cytochrome c binding levels was observed between Δ*rmlACBD*, Δ*rmlT* and wild type EGD-e strains (Fig 4C), indicating that the absence of l-rhamnose in WTAs does not affect the *Lm* surface charge. This was further corroborated by zeta potential measurements showing similar pH-dependent variations for both wild type and mutant strains (S5 Fig in S1 Text). Overall, these results allowed us to discard electrostatic changes as a reason behind the difference in the levels of CRAMP detected at the *Lm* cell surface.

To further explore the decreased levels of surface-exposed CRAMP in *Lm* strains lacking l-rhamnosylated WTAs, we compared total levels of bacterium-associated CRAMP in the different strains by flow cytometry, following a short incubation with a fluorescently labeled form of this AMP. The intensity of *Lm*-associated CRAMP fluorescence was comparable for the wild type EGD-e, Δ*rmlACBD* and Δ*rmlT* strains (Fig 4D and 4E), indicating that the overall peptide levels associated to *Lm* cells were similar between the different strains. Accordingly, the residual fluorescence in the supernatants obtained by centrifugation of the bacteria-peptide suspensions was also similar (Fig 4F). As positive control we used the Δ*dltA* strain, which displayed a significantly stronger peptide binding than its parental wild type strain (Fig 4D–4F). These data strongly suggest that the increased CRAMP susceptibility of *Lm* strains lacking l-rhamnosylated WTAs results from an improved penetration of CRAMP through their cell walls. Altogether, these results showed that l-rhamnosylated WTAs do not interfere with the *Lm* surface charge or with the binding efficiency of AMPs, but likely promote *Lm* survival by hindering the crossing of its cell wall by these bactericidal molecules.

### WTA l-rhamnosylation delays AMP interaction with the *Lm* plasma membrane

**Fig 5:**
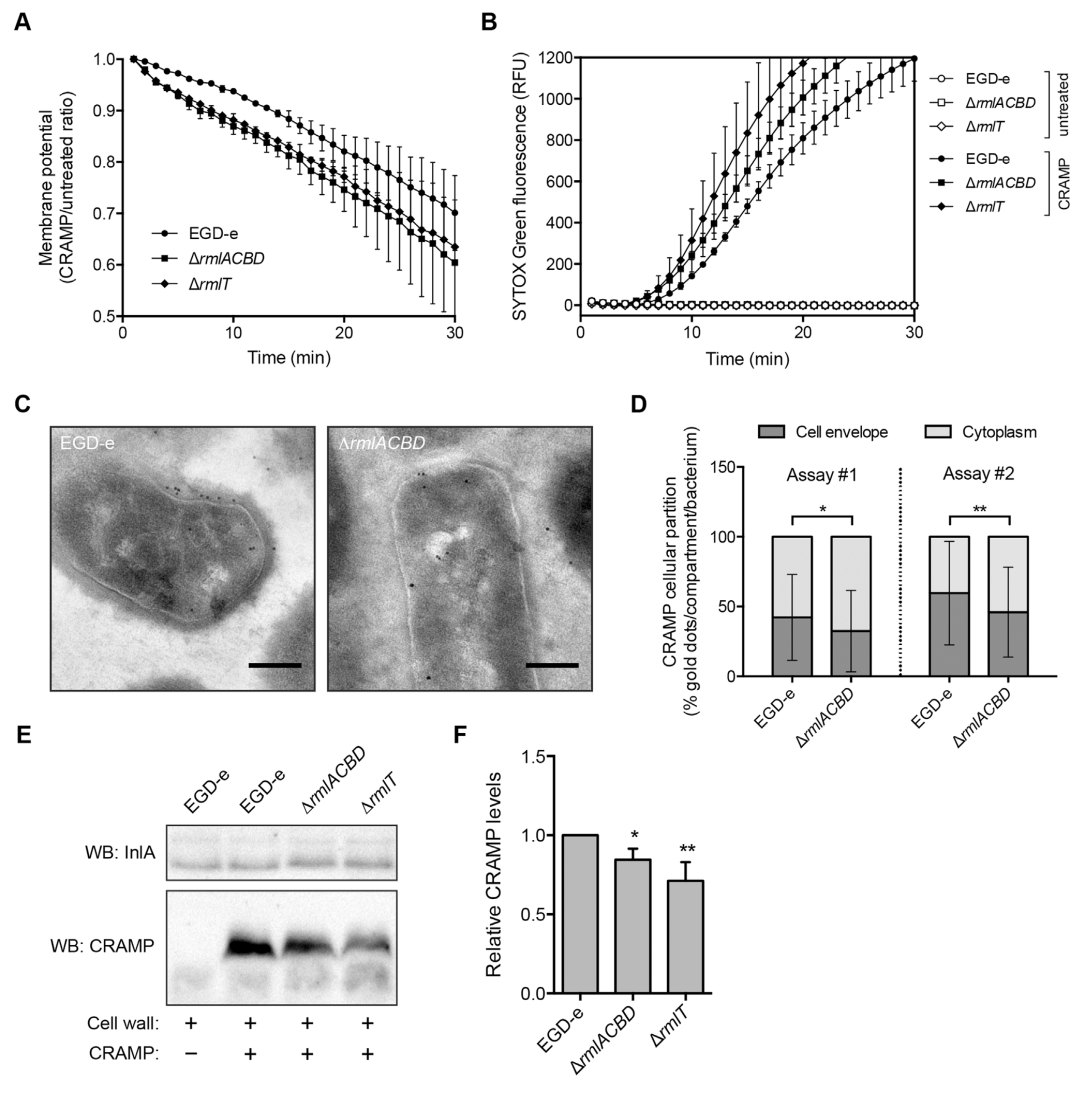
WTA l-rhamnosylation delays AMP interaction with the *Lm* plasma membrane. (**A**) Depolarization rate of *Lm* strains in response to CRAMP. Mid-exponential-phase bacteria pre-stained (15 min) with 30 μM DiOC_2_(3) were challenged with 50 μg/ml CRAMP and changes in the membrane potential, expressed as the ratio of CRAMP-treated versus untreated samples, were monitored during 30 min. Data represent the mean±SD of three independent experiments. (**B**) SYTOX Green uptake kinetics of *Lm* strains in response to CRAMP-mediated membrane permeabilization. Exponential-phase bacteria were incubated (37°C) with PBS (white symbols) or 50 μg/ml CRAMP (black symbols), in the presence of 1 μM SYTOX Green, and the increase in green fluorescence emission was recorded over time. (**C and D**) Transmission electron microscopy analysis of the subcellular distribution of CRAMP in immunogold-labeled sections of mid-exponential-phase wild type and Δ*rmlACBD Lm* strains treated with 50 μg/ml CRAMP (15 min, 37°C). (C) Representative images of contrasted sections of *Lm* cells showing CRAMP-specific gold labeling (10-nm black dots). Scale bar: 0.2 μm. (D) Quantification of the subcellular partition of CRAMP labeling in wild type and Δ*rmlACBD Lm* strains, for two independent assays. The percentages of cell envelope- and cytoplasm-associated gold dots per bacterium were quantified (at least 90 cells per strain) and the results expressed for each strain as mean±SD. (**E and F**) Western blot analysis of levels of CRAMP bound to purified cell wall of different *Lm* strains. Purified cell wall (100 μg) was incubated with CRAMP (5 min), washed and digested overnight with mutanolysin. (E) Supernatants from mutanolysin-treated samples were resolved in 16% Tris-tricine SDS-PAGE and immunoblotted for CRAMP. The *Lm* cell wall-anchored protein InlA was used as loading control. (**F**) Quantification of the relative CRAMP levels represented as the mean±SD of four independent blots. *, *p*≤0.05; **, *p*≤0.01.

In light of these results, we then examined whether WTA l-rhamnosylation interfered with the dynamics of AMP interaction with the *Lm* plasma membrane. We performed a time-course study to follow *Lm* membrane potential changes induced by CRAMP. In live bacteria, the membrane potential is an electric potential generated across the plasma membrane by the concentration gradients of sodium, potassium and chloride ions. Physical or chemical disruption of the plasma membrane integrity leads to the suppression of this potential (depolarization) [48]. *Lm* strains were incubated with DiOC_2_(3), a green fluorescent voltage-sensitive dye that readily enters into bacterial cells. As the intracellular dye concentration increases with higher membrane potential, it favors the formation of dye aggregates that shift the fluorescence emission to red. After stabilization of the DiOC_2_(3) fluorescence, CRAMP was added to bacterial samples and the rate of *Lm* depolarization was immediately analyzed by measuring the red fluorescence emission decline in a flow cytometer. The decrease in the membrane potential was consistently greater in the Δ*rmlACBD* and Δ*rmlT* strains as compared to wild type *Lm*, particularly in the first 10–15 min (Fig 5A), indicating that the *Lm* plasma membrane integrity is compromised faster by the action of CRAMP in the absence of l-rhamnosylated WTAs. To investigate if increased CRAMP-mediated disruption of the *Lm* membrane integrity was associated with increased permeabilization, we monitored in real time the entry of the fluorescent probe SYTOX Green into the different *Lm* strains, following the addition of CRAMP. This probe only enters into bacterial cells with a compromised membrane and displays a strong green fluorescence emission after binding to nucleic acids. As expected, when CRAMP was omitted from the bacterial suspensions, any increase in SYTOX Green-associated fluorescence was detected (Fig 5B). However, in the presence of the peptide, the green fluorescence intensity of samples containing the Δ*rmlACBD* or Δ*rmlT* mutants increased earlier than in samples containing wild type *Lm* (Fig 5B), eventually reaching similar steady-state levels at later time points (S7 Fig in S1 Text). These observations indicate that the CRAMP-mediated permeability increase of the *Lm* membrane to SYTOX Green occurs faster in strains lacking l-rhamnosylated WTAs.

To investigate the ultrastructural localization of the peptide, we performed immunoelectron microscopy on CRAMP-treated wild type and Δ*rmlACBD Lm* strains. Interestingly, CRAMP-specific labeling was not only detected in the *Lm* cell envelope, as expected, but also in the cytoplasm (Fig 5C), suggesting that this AMP may additionally target components or processes inside *Lm*. Comparison of the subcellular distribution of CRAMP between these two bacterial compartments revealed a preferential cell envelope localization in wild type *Lm*, which contrasted with the slight but significantly higher cytoplasmic localization of the peptide in the Δ*rmlACBD* strain (Fig 5D). These observations are in agreement with a model in which CRAMP crosses the *Lm* cell wall more efficiently in the absence of WTA l-rhamnosylation, therefore reaching the bacterial membrane and the cytoplasm comparatively faster.

Finally, to confirm that the presence of l-rhamnosylated WTAs hinders the capacity of AMPs to flow through the *Lm* cell wall, we assessed levels of CRAMP retained in purified cell wall samples from the wild type, Δ*rmlACBD* and Δ*rmlT* strains by Western blot. After incubation with CRAMP, peptides trapped within the peptidoglycan matrix were released by mutanolysin treatment of the cell wall and quantitatively resolved by SDS-PAGE. Immunoblotting revealed a small but consistent decrease in the amount of peptide associated with the cell wall from the two mutant strains in comparison with wild type *Lm* (Fig 5E and 5F). This result indicates that the lack of l-rhamnose in WTAs results in a partial loss of the AMP retention capacity of the *Lm* cell wall, which induces an enhanced AMP targeting of the *Lm* plasma membrane and consequent bacterial killing.

All combined, these data support a model where the l-rhamnosylation of WTAs alters the *Lm* cell wall permeability to favor the entrapment of AMPs. This obstructive effect hinders AMP progression through the cell wall and delays their lethal interaction with the plasma membrane.

### WTA l-rhamnosylation is crucial for AMP resistance *in vivo* and *Lm* virulence

**Fig 6:**
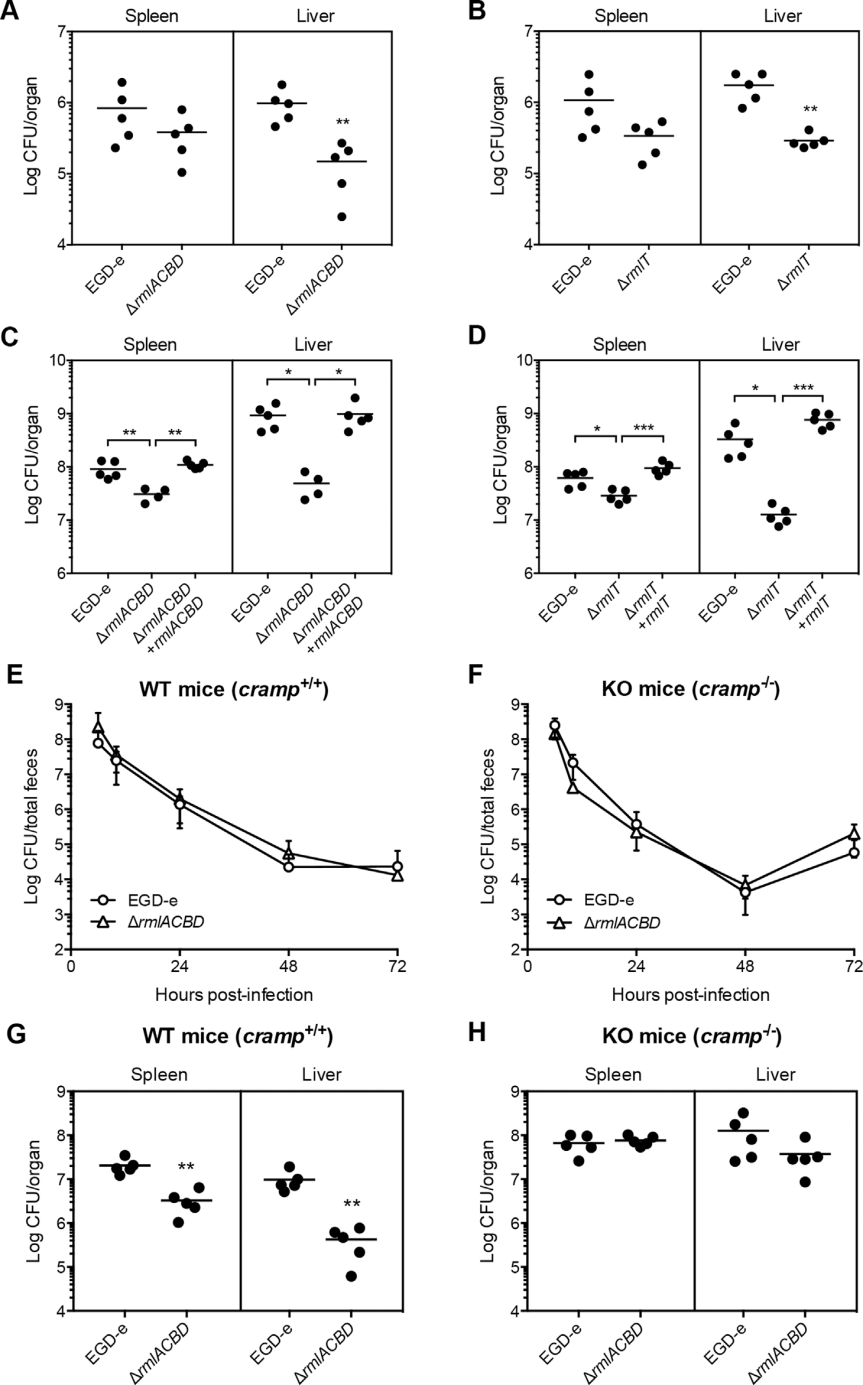
WTA l-rhamnosylation is necessary for AMP resistance *in vivo* and *Lm* virulence. (**A–D**) Quantification of viable bacteria in the spleen and liver recovered from BALB/c mice (n = 5), three days after (A and B) oral or (C and D) intravenous infection with sub-lethal doses of indicated *Lm* strains. Data are presented as scatter plots, with each animal indicated by a dot and the mean indicated by a horizontal line. **(E and F**) Quantification of the fecal shedding of wild type or Δ*rmlACBD Lm* strains after oral infection of (E) wild type (WT, *cramp* ^+/+^) and (F) CRAMP knockout (KO, *cramp* ^-/-^) 129/SvJ mice (n = 5). Total feces produced by each animal at specific time points were collected and processed for bacterial enumeration in *Listeria*-selective agar media. Data are expressed as mean±SD. (**G and H**) Quantification of viable bacteria in spleens and livers recovered from (G) wild type (WT, *cramp* ^+/+^) and (H) CRAMP knockout (KO, *cramp* ^-/-^) 129/Sv mice (n = 5), three days after intravenous infection with sub-lethal doses of wild type or Δ*rmlACBD Lm* strains. Data are presented as scatter plots, with each animal represented by a dot and the mean indicated by a horizontal line. *, *p*≤0.05; **, *p*≤0.01; ***, *p*≤0.001.

To evaluate the importance of WTA l-rhamnosylation in *Lm* pathogenicity, we assessed the *in vivo* virulence of *Lm* strains lacking l-rhamnosylated WTAs. BALB/c mice were inoculated orally with wild type, Δ*rmlACBD* or Δ*rmlT* strains, and the bacterial load in the spleen and liver of each animal was quantified three days later. The proliferative capacity of both Δ*rmlACBD* and Δ*rmlT* mutant strains was similarly reduced in both organs, although more significantly in the liver (Fig 6A and 6B). To determine if the decreased virulence of the mutant strains was due to a specific defect in the crossing of the intestinal epithelium, BALB/c mice were challenged intravenously, bypassing the intestinal barrier. Three days post-infection, the differences between mutant and wild type strains, in both organs, were similar to those observed in orally infected animals (Fig 6C and 6D), thus discarding any sieving effect of the intestinal epithelium on the decreased splenic and hepatic colonization by both Δ*rmlACBD* and Δ*rmlT*. Importantly, organs of mice infected intravenously with the complemented strains (Δ*rmlACBD*+*rmlACBD* and Δ*rmlT*+*rmlT*) displayed bacterial loads comparable to wild type *Lm*-infected organs (Fig 6C and 6D). The attenuated *in vivo* phenotype of the Δ*rmlACBD* and Δ*rmlT* strains was not caused by an intrinsic growth defect, as demonstrated by their wild type-like growth profiles in broth or inside eukaryotic cells (S8 Fig in S1 Text). These results confirmed the involvement of the *rml* operon in virulence, revealing a significant contribution of WTA l-rhamnosylation to *Lm* pathogenesis. Importantly, the *in vivo* attenuation of the Δ*rmlT* strain, which is unable to append l-rhamnose to its WTAs but is able to synthesize the l-rhamnose precursor, showed that although l-rhamnose biosynthesis is required to achieve optimal levels of virulence it is its covalent linkage to the WTA backbone that is crucial for the successful *Lm* host infection.

To evaluate the protective role of WTA l-rhamnosylation against AMPs *in vivo*, we performed virulence studies in a CRAMP-deficient mouse model. To determine the influence of WTA l-rhamnosylation in *Lm* intestinal persistence, we performed oral infections of adult CRAMP knockout 129/SvJ mice (*cramp* ^-/-^, KO) [49] and of age- and background-matched wild type mice (*cramp* ^+/+^, WT), with the wild type or Δ*rmlACBD Lm* strains and monitored the respective fecal carriage. In both WT and KO mice, we observed comparable dynamics of fecal shedding of the wild type and Δ*rmlACBD* strains (Fig 6E and 6F). In agreement with the comparable virulence defects observed for WTA l-rhamnosylation-deficient bacteria, following oral or intravenous inoculation of BALB/c mice (Fig 6A–6D), these results suggest a minor role for CRAMP in the control of *Lm* during the intestinal phase of the infection.

We then inoculated WT and KO mice intravenously and quantified bacterial numbers in the spleen and liver, three days post-infection. In line with what was observed in BALB/c mice (Fig 6C), the Δ*rmlACBD* strain showed significant virulence attenuation in both organs of WT mice (Fig 6G). Interestingly, this virulence defect was nearly abolished in KO animals, with the Δ*rmlACBD* strain displaying an organ-colonizing capacity similar to wild type bacteria (Fig 6H). In addition, bacterial loads were higher in the organs of KO mice than in those of WT animals (Fig 6G and 6H). These data indicate that, in comparison to their WT congeners, KO mice are more susceptible to *Lm* infection, and confirm the *in vivo* listericidal activity of CRAMP.

Altogether, these results highlight a key role for host-produced CRAMP in restraining *Lm* infection and demonstrate that WTA l-rhamnosylation also promotes resistance to AMPs in an *in vivo* context.

## Discussion

Teichoic acids are key players in the maintenance of the Gram-positive cell envelope integrity and functionality. They are typically decorated with d-alanine and/or a variety of glycosyl groups, which influence the overall properties of these polymers [9]. Whereas d-alanylation of WTAs has been demonstrated to contribute towards bacterial defense against AMPs [14, 23], the involvement of glycosylation in this process has never been investigated. In this study, we show for the first time that the glycosylation of *Lm* WTAs with l-rhamnose is mediated by the WTA l-rhamnosyltransferase RmlT and confers protection against AMPs *in vitro* and during mouse infection. Based on our data, we propose that this protection results from a delayed traversal of the *Lm* cell envelope by AMPs in the presence of l-rhamnose-decorated WTAs. Most importantly, we reveal a key role for l-rhamnosylated WTAs in the processes underlying *Lm* pathogenesis.

Unlike *S*. *aureus* or *B*. *subtilis* [22], WTAs in *Listeria* are not decorated with d-alanine, undergoing only glycosylation with a small pool of monosaccharides [6, 10]. Among these is l-rhamnose, which is the product of a remarkably conserved biosynthetic pathway that is encoded by the *rmlABCD* genes [35]. Interestingly, a significant number of bacteria harboring these genes are commonly pathogenic [27–32] and have l-rhamnose in close association with surface components [50, 51]. In *Listeria*, the *rmlACBD* locus is only found in certain serotypes of *Lm* (1/2a, 1/2b, 1/2c, 3c and 7) and *L*. *seeligeri* (1/2b). These serotypes were all shown to have l-rhamnose in their WTAs, except for *Lm* serotypes 3c and 7 [6], which appear to be unable to produce this sugar because of mutations within *rmlA* and *rmlB*, respectively (Fig 1). Our results confirmed that the appendage of l-rhamnose to *Lm* WTAs requires the products of the *rmlACBD* locus. Ultimately, WTA glycosylation is catalyzed by glycosyltransferases, a class of enzymes that recognize nucleotide-sugar substrates and transfer the glycosyl moiety to a WTA subunit [52]. *In silico* analysis of *lmo1080*, the first gene of the operon including *rmlACBD* (Fig 1) showed that it encodes a protein with putative glycosyltransferase activity. The genomic location and predicted protein function were strong indicators that this gene might encode the transferase involved in the l-rhamnosylation of *Lm* WTAs. Our data demonstrated that whereas *lmo1080*, that we renamed *rmlT*, is dispensable for rhamnose biosynthesis, it is required for the addition of l-rhamnose to WTAs in *Lm* strains with a functional l-rhamnose pathway, thus validating RmlT as the l-rhamnose-specific WTA glycosyltransferase in *Lm*.

WTAs are associated with the natural resistance of *S*. *aureus* to peptidoglycan-degrading enzymes, such as lysozyme [37, 41]. In contrast, absence of WTA decoration, but not of the polymers, was shown to induce an increase of the staphylococcal susceptibility to lysostaphin [53]. Modifications of the *Lm* peptidoglycan, such as *N*-deacetylation [42], were found to contribute to protection against lysozyme, but the role of WTAs and in particular their decoration, was never addressed. Our results discard WTA l-rhamnosylation as a component of the *Lm* resistance mechanism to this host immune defense protein, as well as its involvement in the promotion of growth under osmotic conditions. Other innate immune effectors, such as antimicrobial peptides (AMPs), also target bacterial organisms [54] that in turn have developed resistance strategies to avoid injury and killing induced by AMPs. Among these strategies is the reshaping and fine-tuning of cell envelope components to lower AMP affinity to the bacterial surface [21]. Previous studies showed a clear link between the d-alanylation of WTAs and AMP resistance [14, 43]. In this context, we found here a similar role for WTA l-rhamnosylation, showing that, in the absence of l-rhamnosylated WTAs, bacteria exhibit an increased susceptibility to AMPs produced by bacteria, mice and importantly by humans. Although from such distinct sources, AMPs used here share a cationic nature that supports their activity. However, while teichoic acid d-alanylation is known to reduce the cell wall electronegativity [14], glycosyl substituents of *Lm* WTAs are neutrally charged and WTA glycosylation should thus promote AMP resistance through a different mechanism.

It is well established that AMPs induce bacterial death mainly by tampering with the integrity of the plasma membrane. This can be achieved through multiple ways, all of which are driven by the intrinsic amphipathic properties of this class of peptides [55]. Nonetheless, the initial interaction of AMPs with bacterial surfaces is mediated by electrostatic forces between their positive net charge and the anionic cell envelope [23]. Our data show that, unlike d-alanylation [56], WTA l-rhamnosylation does not interfere with the *Lm* cell surface charge, in agreement with l-rhamnose being an electrostatically neutral monosaccharide. Importantly, the reduced levels of surface-exposed CRAMP in *Lm* strains lacking l-rhamnosylated WTAs suggested instead that their increased susceptibility to this peptide was correlated with its improved penetration of the l-rhamnose-depleted *Lm* cell wall. We confirmed this premise with data showing that CRAMP-mediated cell depolarization and plasma membrane permeabilization events occur earlier in WTA l-rhamnosylation-deficient *Lm* strains. In addition, we also observed a predominant cytoplasmic presence of CRAMP in these mutant strains, in contrast to the preferential cell envelope localization in wild type *Lm*, further suggesting a WTA l-rhamnosylation-dependent kinetic discrepancy in the progression of CRAMP through the *Lm* cell envelope. Saar-Dover *et al*. demonstrated in the WTA-lacking *Streptococcus agalactiae* (GBS) that LTA d-alanylation promoted resistance to the human cathelicidin LL-37 by hindering cell wall crossing and plasma membrane disturbance [57]. They proposed that the underlying mechanism does not rely on modulation of the surface charge but on LTA conformation-associated alterations of the cell wall packing density [57]. Our data are in line with these observations and although we did not detect changes in the cell wall cross-linking status, we cannot ignore a possible impact of l-rhamnosylation on WTA polymer conformation accounting for changes in cell wall permeability. If one considers that the peptidoglycan, a multi-layered and compact structure, is densely populated with WTA polymers decorated with multiple units of the rather bulky l-rhamnose molecule, spatial constraints and increased cell wall density need to be accounted. In fact, we showed that purified *Lm* cell wall depleted of l-rhamnose does not retain CRAMP in its peptidoglycan matrix as effectively as cell wall containing l-rhamnosylated WTAs. In addition, we have indications that soluble l-rhamnose interferes with CRAMP activity, improving the survival of WTA l-rhamnosylation mutants of *Lm*. These observations suggest a potential interaction between l-rhamnose and AMPs, which could favor the “retardation effect” that ultimately promotes *Lm* survival.

We previously reported a significantly increased transcription of *rmlACBD* during mouse spleen infection [24], which suggested that WTA l-rhamnosylation is highly activated by *Lm* to successfully infect this host organ. Our infection studies in mice confirmed the importance of this mechanism for *Lm* pathogenesis by revealing a significant virulence attenuation of WTA l-rhamnosylation-deficient *Lm* strains. Surprisingly, the expression of *rmlT* appeared unchanged during mouse spleen infection as compared to growth in BHI [24], suggesting that an increased L-rhamnose biosynthesis could be sufficient to induce an increased WTA l-rhamnosylation and AMP resistance. Faith *et al*. also observed a decreased bacterial burden of a serotype 4b *Lm* strain lacking the *gtcA* gene [58], a mutation that resulted in complete loss of galactose decoration of its WTAs [59]. Interestingly, *gtcA* is also present in *Lm* EGD-e, where it appears to be involved in WTA substitution with *N*-acetylglucosamine [60], and was shown to contribute to the colonization of the mouse spleen, liver and brain [61]. However the mechanism through which this occurs remains unclear.

Virulence studies in mice lacking the CRAMP gene corroborated our *in vitro* susceptibility data and revealed the importance of WTA l-rhamnosylation-promoted resistance to AMPs for *Listeria* virulence. *In vivo* data also provided a strong insight into the protective role of CRAMP against systemic infection by *Lm*, as had been previously observed with other bacterial pathogens [49, 62, 63]. Our results on fecal shedding dynamics suggest that the contribution of CRAMP to the control of *Lm* during the intestinal phase of infection is minimal. A previous report showed a negligible enteric secretion of CRAMP in normal adult mice [64], which may explain the similar shedding behavior of the wild type and Δ*rmlACBD* strains that were observed in both mouse strains. In this scenario, infection studies in newborn animals, whose enterocytes actively express CRAMP [45, 64], may provide conclusive information regarding the role of WTA l-rhamnosylation in the *Lm* resistance to CRAMP during the intestinal phase of the infection. Notwithstanding, CRAMP is actively produced by phagocytes in adult mice [65]. As a major target for *Lm* colonization, the spleen is also an important reservoir of phagocytic cells. We can speculate that WTA l-rhamnosylation is particularly important to increase the chances of *Lm* surviving CRAMP-mediated killing during spleen infection. Considering our data on the *Lm* susceptibility to LL-37, the human homolog of CRAMP, we can also envisage this scenario in the context of human infection.

In conclusion, our work has unveiled for the first time a role for WTA glycosylation in bacterial resistance to AMPs. We propose that WTA l-rhamnosylation reduces the cell wall permeability to AMPs, promoting a delay in the crossing of this barrier and in the disruption of the plasma membrane, thus favoring *Lm* survival and virulence *in vivo*. Our findings reveal a novel facet in the contribution of WTA modifications towards AMP resistance, reinforcing the crucial role of these Gram-positive surface glycopolymers in host defense evasion.

## Materials and Methods

### Bacterial strains and growth conditions

Bacterial strains used in this study are listed in Table 1. *Lm* and *E*. *coli* strains were routinely cultured aerobically at 37°C in brain heart infusion (BHI, Difco) and Lysogeny Broth (LB) media, respectively, with shaking. For experiments involving the *Lm* Δ*tagO1*Δ*tagO2* strain, bacteria were first cultured overnight at 30°C with shaking in the presence of 1 mM IPTG (isopropyl-β-d-thiogalactopyranoside), washed and diluted (1:100) in fresh BHI and cultured overnight at 30°C with shaking [36]. When appropriate, the following antibiotics were included in culture media as selective agents: ampicilin (Amp), 100 μg/ml; chloramphenicol (Cm), 7 μg/ml (*Lm*) or 20 μg/ml (*E*. *coli*); erythromycin (Ery), 5 μg/ml. For genetic complementation purposes, colistin sulfate (Col) and nalidixic acid (Nax) were used at 10 and 50 μg/ml, respectively.

**Table 1:** Plasmids and bacterial strains.

Plasmid or strain	Code	Relevant characteristics	Source
Plasmids			
pMAD		Gram-negative/Gram-positive shuttle vector; thermosensitive replication; Amp^r^ Ery^r^	[66]
pPL2		*L*. *monocytogenes* phage-derived site-specific integration vector; Cm^r^	[67]
pMAD(Δ*rmlACBD*)	pDC303	pMAD with 5’- and 3’-flanking regions of *rmlACBD* locus; Amp^r^ Ery^r^	This study
pPL2(*rmlACBD*)	pDC313	pPL2 with *rmlACBD* locus and 5’- and 3’-flanking regions; Cm^r^	This study
pMAD(Δ*rmlACBD*)	pDC491	pMAD with 5’- and 3’-flanking regions of *rmlT*; Amp^r^ Ery^r^	This study
pPL2(*rmlT*)	pDC550	pPL2 with *rmlT* sequence and 5’- and 3’-flanking regions; Cm^r^	This study
*E*. *coli* strains			
DH5α		Cloning host strain; F^-^ Φ80*lac*ZΔM15 Δ(*lacZYA*-*argF*) U169 *recA1 endA1 hsdR17*(r_k_^-^, m_k_^+^) *phoA supE44 thi-1 gyrA96 relA1* λ^-^	Life Technologies
S17-1		Conjugative donor strain; *rec*A *pro hsdR* RP4-2-Tc::Mu-Km::Tn7	[77]
*L*. *monocytogenes* strains			
EGD-e		wild type; serotype 1/2a	[78]
EGD-e Δ*pgdA*		EGD-e *pgdA* (*lmo0415*) deletion mutant	[42]
EGD-e Δ*rmlACBD*	DC307	EGD-e *rmlACBD* (*lmo1081–4*) deletion mutant	This study
EGD-e Δ*rmlACBD*::pPL2(*rmlACBD*)	DC367	EGD-e *rmlACBD* (*lmo1081–4*) deletion mutant complemented with pPL2(*rmlACBD*) (pDC313); Cm^r^	This study
EGD-e Δ*rmlT*	DC492	EGD-e *rmlT* (*lmo1080*) deletion mutant	This study
EGD-e Δ*rmlT*::pPL2(*rmlT*)	DC553	EGD-e *rmlT* (*lmo1080*) deletion mutant complemented with pPL2(*rmlT*) (pDC550); Cm^r^	This study
EGD-e Δ*tagO1*Δ*tagO2*::pLIV2(*tagO1*)		EGD-e *tagO1* (*lmo0959*) and *tagO2* (*lmo2519*) double deletion mutant complemented with pLIV2(*tagO1*), expressing *tagO1* under the control of an IPTG-inducible promoter; Cm^r^	[36]
EGD	BUG600	wild type; serotype 1/2a	[79]
EGD Δ*dltA*	BUG2182	EGD *dltA* (*LMON_0982*) deletion mutant	[80]

### Construction and complementation of mutant strains

*Lm* mutant strains were constructed in the EGD-e background through a process of double homologous recombination mediated by the suicide plasmid pMAD [66]. DNA fragments corresponding to the 5’- and 3’-flanking regions of the *rmlACBD* locus (*lmo1081—4*) were amplified by PCR from *Lm* EGD-e chromosomal DNA with primers 1–2 and 3–4 (S2 Table in S1 Text), and cloned between the *Sal*I—*Mlu*I and *Mlu*I—*Bgl*II sites of pMAD, yielding pDC303. Similarly, DNA fragments corresponding to the 5’- and 3’-flanking regions of *rmlT* (*lmo1080*) were amplified with primers 15–16 and 17–18 (S2 Table in S1 Text), and cloned between the *Sal*I—*Eco*RI and *Eco*RI—*Bgl*II sites of pMAD, yielding pDC491. The plasmid constructs were introduced in *Lm* EGD-e by electroporation and transformants selected at 30°C in BHI—Ery. Positive clones were re-isolated in the same medium and grown overnight at 43°C. Integrant clones were inoculated in BHI broth and grown overnight at 30°C, after which the cultures were serially diluted, plated in BHI agar and incubated overnight at 37°C. Individual colonies were tested for growth in BHI—Ery at 30°C and antibiotic-sensitive clones were screened by PCR for deletion of *rmlACBD* (primers 5–6, 7–8, 9–10 and 11–12) and *rmlT* (primers 19–20) (S2 Table in S1 Text). Genetic complementation of the deletion mutant strains was performed as described [24]. DNA fragments containing either the *rmlACBD* or *rmlT* loci were amplified from *Lm* EGD-e chromosomal DNA with primers 13–14 and 21–22 (S2 Table in S1 Text), respectively, and cloned between the *Sal*I—*Pst*I sites of the phage-derived integrative plasmid pPL2 [67], generating pDC313 and pDC550. The plasmid constructs were introduced in the *E*. *coli* strain S17-1 and transferred, respectively, to the Δ*rmlACBD* and Δ*rmlT* strains by conjugation on BHI agar. Transconjugant clones were selected in BHI—Cm/Col/Nax and chromosomal integration of the plasmids confirmed by PCR with primers 23 and 24 (S2 Table in S1 Text). All plasmid constructs and gene deletions were confirmed by DNA sequencing.

### Gene expression analyses

Total bacterial RNA was isolated from 10 ml of exponential cultures (OD_600_ = 0.6) by the phenol-chloroform extraction method, as previously described [68], and treated with DNase I (Turbo DNA-free, Ambion), as recommended by the manufacturer. Purified RNAs (1 μg) were reverse-transcribed with random hexamers, using iScript cDNA Synthesis kit (Bio-Rad Laboratories). Quantitative real-time PCR (qPCR) was performed in 20-μl reactions containing 2 μl of cDNA, 10 μl of SYBR Green Supermix (Bio-Rad Laboratories) and 0.25 μM of forward and reverse primers (S2 Table in S1 Text), using the following cycling protocol: 1cycle at 95°C (3 min) and 40 cycles at 95°C (30 s), 55°C (30 s) and 72°C (30 s). Each target gene was analyzed in triplicate and blank (water) and DNA contamination controls (unconverted DNase I-treated RNA) were included for each primer pair. Amplification data were analyzed by the comparative threshold (ΔΔCt) method, after normalization of the test and control sample expression values to a housekeeping gene (16S rRNA). For qualitative analysis, PCR was performed in 20-μl reactions containing 2 μl of cDNA, 10 μl of MangoMix 2× reaction mix (Bioline) and 0.5 μM of forward and reverse qPCR primers, using the following protocol: 1 cycle at 95°C (5 min), 25 cycles at 95°C (30 s), 55°C (30 s) and 72°C (20 s), and 1 cycle at 72°C (5 min). Amplification products were resolved in 1% (w/v) agarose gel and analyzed in a GelDoc XR+ System (Bio-Rad Laboratories).

### WTA PAGE analysis

Extraction and analysis of *Lm* WTAs by polyacrylamide gel electrophoresis was performed essentially as described [69], with the exception that WTAs extracts were obtained from exponential-phase cultures. Sedimented bacteria were washed (buffer 1: 50 mM MES buffer, pH 6.5) and boiled for 1 h (buffer 2: 4% SDS in buffer 1). After centrifugation, the pellet was serially washed with buffer 2, buffer 3 (2% NaCl in buffer 1) and buffer 1, before treatment with 20 μg/ml proteinase K (20 mM Tris-HCl, pH 8; 0.5% SDS) at 50°C for 4 h. The digested samples were thoroughly washed with buffer 3 and distilled water and incubated overnight (16 h) with 0.1 M NaOH, under vigorous agitation. Cell wall debris were removed by centrifugation (10,000 rpm, 10 min) and the hydrolyzed WTAs present in the supernatant were directly analyzed by native PAGE in a Tris-tricine buffer system. WTA extracts were resolved through a vertical (20 cm) polyacrylamide (20%) gel at 20 mA for 18 h (4°C). To visualize WTAs, the gel was stained in 0.1% Alcian blue (40% ethanol; 5% acetic acid) for 30 min and washed (40% ethanol; 10% acetic acid) until the background is fully cleared. Optionally, for increased contrasting, silver staining can be performed on top of the Alcian blue staining.

### Purification of cell wall components

Cell walls of *Lm* strains were purified as described before [70], with modifications. Overnight cultures were subcultured into 1–2 liters of BHI broth (initial OD_600_ = 0.005) and bacteria grown until exponential phase (OD_600_ = 1.0–1.5). Cultures were rapidly cooled in an ice/ethanol bath and bacteria harvested by centrifugation (7,500 rpm, 15 min, 4°C). The pellet was resuspended in cold ultrapure water and boiled for 30 min with 4% SDS to kill bacteria and inactivate cell wall-modifying enzymes. The samples were cleared of SDS by successive cycles of centrifugation (12,000 rpm, 10 min) and washing with warm ultrapure water until no detergent was detected [71]. SDS-free samples were resuspended in 2 ml of ultrapure water and cell walls disrupted with glass beads in a homogenizer (FastPrep, Thermo Savant). Fully broken cell walls were separated from glass beads by filtration (glass filters, pore size: 16–40 μm) and from unbroken cell walls and other debris by low-speed centrifugation (2,000 rpm, 15 min). Nucleic acids were degraded after incubation (2 h) at 37°C with DNase (10 μg/ml) and RNase (50 μg/ml) in a buffer containing 50 mM Tris-HCl, pH 7.0, and 20 mM MgSO_4_. Proteins were then digested overnight at 37°C with trypsin (100 μg/ml) in the presence of 10 mM CaCl_2_. Nuclease and proteases were inactivated by boiling in 1% SDS, and samples were centrifuged (17,000 rpm, 15 min) and washed twice with ultrapure water. Cell walls were resuspended and incubated (37°C, 15 min) in 8 M LiCl and then in 100 mM EDTA, pH 7.0, after which they were washed twice with water. After resuspension in acetone and sonication (15 min), cell walls were washed and resuspended in ultrapure water before undergoing lyophilization.

To obtain purified peptidoglycan, cell walls (20 mg) were incubated for 48 h with 4 ml of 46% hydrofluoric acid (HF), under agitation at 4°C. Samples were washed with 100 mM Tris-HCl, pH 7.0, and centrifuged (17,000 rpm, 30 min, 4°C) as many times as necessary to neutralize the pH. The pellet was finally washed twice with water prior to lyophilization. WTA extracts were obtained by incubating 1 mg of cell wall with 300 μl of 46% HF (18 h, 4°C). After centrifugation (13,200 rpm, 15 min, 4°C), the supernatant was recovered and evaporated under a stream of compressed air. The dried WTA residue was resuspended in water and lyophilized.

### Extraction of bacterial cytoplasmic content

The intracellular content of *Lm* strains was isolated according to a modified version of the protocol by Ornelas-Soares *et al*. [72]. Bacterial cultures (200 ml) were grown until early exponential phase (OD_600_ = 0.3), and vancomycin was added at 7.5 μg/ml (5×MIC value [73]) to induce the cytoplasmic accumulation of the peptidoglycan precursor UDP-MurNAc-pentapeptide. Cultures were grown for another 45 min and chilled in an ice-ethanol bath for 10 min. Bacteria were then harvested by centrifugation (12,000 rpm, 10 min, 4°C), washed with cold 0.9% NaCl, resuspended in 5 ml of cold 5% trichloroacetic acid and incubated for 30 min on ice. Cells and other debris were separated by centrifugation (4,000 rpm, 15 min, 4°C) and the supernatant was extracted with 1–2 volumes of diethyl ether as many times as necessary to remove TCA (sample pH should rise to at least 6.0). The aqueous fraction containing the cytoplasmic material was lyophilized and the dried residue resuspended in ultrapure water.

### HPLC analyses

To analyze their sugar composition, purified cell wall and peptidoglycan (200 μg each), as well as cytoplasmic (500 μg) and WTA extracts were hydrolyzed in 3 M HCl for 2 h at 95°C. After vacuum evaporation, the samples were washed with water and lyophilized. The hydrolyzed material was then resuspended in 150 μl of water and resolved by high-performance anion-exchange chromatography coupled with pulsed amperometric detection (HPAEC-PAD). Ten microliters were injected into a CarboPac PA10 column (Dionex, Thermo Fisher Scientific) and eluted at 1 ml/min (30°C) with 18 mM NaOH, followed by a gradient of NaCH_3_COO: 0–20 mM (t = 25–30 min), 20–80 mM (t = 30–35 min), 80–0 mM (t = 40–45 min). Standards for glucosamine, muramic acid, l-rhamnose and ribitol (Sigma-Aldrich) were eluted under the same conditions to enable identification of chromatogram peaks. Data were acquired and analyzed with the Chromeleon software (Dionex, Thermo Fisher Scientific).

Muropeptide samples were prepared and analyzed as described [74], with minor changes. Purified peptidoglycan was digested with 200 μg/ml mutanolysin (Sigma-Aldrich) in 12.5 mM sodium phosphate, pH 5.5, for 16 h at 37°C. Enzymatic activity was halted by heating at 100°C for 5 min, after which the digested sample was reduced for 2 h with 2.5 mg/ml of sodium borohydride (NaBH_4_) in 0.25 M borate buffer, pH 9.0. The reaction was stopped by lowering the sample pH to 2 with ortho-phosphoric acid. After centrifugation, the supernatant was analyzed by reverse phase HPLC. Fifty microliters were injected into a Hypersil ODS (C18) column (Thermo Fisher Scientific) and muropeptide species eluted (0.5 ml/min, 52°C) in 0.1 M sodium phosphate, pH 2.0, with a gradient of 5–30% methanol and detected at 206 nm.

### Intracellular multiplication

Mouse macrophage-like J774A.1 cells (ATCC, TIB-67) were propagated in Dulbecco’s modified Eagle’s medium (DMEM) containing 10% fetal bovine serum and infection assays were performed as described [24]. Briefly, cells (~2×10^5^/well) were infected for 45 min with exponential-phase bacteria at a multiplicity of infection of ~10 and treated afterwards with 20 μg/ml gentamicin for 75 min. At several time-points post-infection, cells were washed with PBS and lysed in cold 0.2% Triton X-100 for quantification of viable intracellular bacteria in BHI agar. One experiment was performed with triplicates for each strain and time-point.

### Resistance to salt stress and lysozyme

*Lm* cultures grown overnight were appropriately diluted in BHI broth and their growth under the presence of stressful stimuli was monitored by optical density measurement at 600 nm (OD_600_). For comparative analysis of *Lm* resistance to salt stress, bacterial cultures were diluted 100-fold in BHI alone (control) or BHI containing 5% NaCl. To assess the *Lm* resistance to lysozyme, exponential-phase cultures (OD_600_ ≈ 1.0) were challenged with different doses of chicken egg white lysozyme (Sigma). A mutant *Lm* strain hypersensitive to lysozyme (Δ*pgdA*) was used as a positive control for susceptibility.

### AMP susceptibility

Bacteria in the exponential phase of growth (OD_600_ = 0.7–0.8) were diluted (10^4^ CFU/ml) in sterile PB medium (10 mM phosphate buffer, pH 7.4; 1% BHI) and mixed in a 96-well microplate with increasing concentrations of gallidermin (Santa Cruz Biotechnology), CRAMP or LL-37 (AnaSpec). Bacterial suspensions without AMPs were used as reference controls for optimal growth/survival. After incubation for 2 h at 37°C, the mixtures were serially diluted in sterile PBS and plated in BHI agar for quantification of viable bacteria. Each condition was analyzed in duplicate in three independent assays.

### Cytochrome c binding

Cytochrome c binding assays were performed as described [56]. Bacteria from mid-exponential-phase cultures (OD_600_ = 0.6–0.7) were washed in 20 mM MOPS buffer, pH 7.0, and resuspended in ½ volume of 0.5 mg/ml equine cytochrome c (Sigma-Aldrich) in 20 mM MOPS buffer, pH 7.0. After 10 min of incubation, bacteria were pelleted and the supernatant collected for quantification of the absorbance at 530 nm. The mean absorbance values from replicate samples containing bacteria were subtracted to the mean value of a reference sample lacking bacteria, and the results were presented for each strain as percentage of unbound cytochrome c.

### Zeta potential measurements

Bacteria (1 ml) from mid-exponential-phase cultures were washed twice with deionized water and diluted (10^7^ CFU/ml) in 15 mM NaCl solutions adjusted to different pH values (1 to 7) with nitric acid. Bacterial suspensions (750 μl) were injected into a disposable capillary cell cuvette (DTS1061, Malvern Instruments) and the zeta potential was measured at 37°C in a ZetaSizer Nano ZS (Malvern Instruments), under an automated field voltage. Samples were measured in triplicate in three independent assays.

### Flow cytometry analyses

Bacteria from 500 μl of mid-exponential-phase cultures were washed twice with PBS and treated for 5 min with 5 μg/ml CRAMP or PBS (untreated control). After centrifugation, the supernatant was removed and PBS-washed bacteria were incubated for 1 h with rabbit anti-CRAMP (1:100, Innovagen), followed by 1 h with Alexa Fluor 488-conjugated anti-rabbit IgG (1:200, Molecular Probes). Finally, bacteria were fixed with 3% paraformaldehyde for 15 min, washed and resuspended in PBS. Alternatively, bacteria were similarly treated with an N-terminally 5-FAM-labeled synthetic form of CRAMP (95% purity, Innovagen), washed and resuspended in PBS. Samples were acquired in a FACSCalibur flow cytometer equipped with CellQuest software (BD Biosciences) and data were analyzed with FlowJo (TreeStar Inc.). Green fluorescence was collected from at least 50,000 FSC/SSC-gated bacterial events in the FL1 channel (530 nm/20 nm bandpass filter). Fluorescence intensities were plotted in single-parameter histograms and results were presented as the average mean fluorescence intensity (MFI) value from three independent analyses.

For bacterial membrane potential studies, the lipophilic fluorescent probe DiOC_2_(3) (3,3-diethyloxacarbocyanine, Santa Cruz Biotechnology) was used as a membrane potential indicator [48, 75]. Mid-logarithmic phase bacteria were diluted (10^6^ CFU/ml) in PBS with 30 μM DiOC_2_(3) and incubated for 15 min in the dark. CRAMP was added to a final concentration of 50 μg/ml and the sample was immediately injected in the flow cytometer. Control samples treated with PBS or with 1.5 mM sodium azide (uncoupling agent) were analyzed to determine the fluorescence values corresponding to basal (100%) and null (0%) membrane potential (S6 Fig in S1 Text). Green and red (FL3, 670 nm/long bandpass filter) fluorescence emissions were continuously collected from FSC/SSC-gated bacteria for 30 min. After acquisition, a ratio of red over green fluorescence (R/G) was calculated per event and plotted in the y-axis versus time. A series of consecutive one-minute-wide gates was applied to the plot and the mean R/G value per gate was determined. The mean R/G values from uncoupler-treated samples were deducted from the corresponding values from the untreated and CRAMP-treated samples, and the resulting values for each condition were normalized as percentage of the initial value (t = 1 min). Finally, the temporal variation of the *Lm* membrane potential was represented graphically as the ratio of the normalized values from CRAMP-treated over untreated samples.

### SYTOX Green uptake

Bacterial uptake of the cell-impermeable SYTOX Green dye was used to study membrane permeabilization induced by CRAMP [57]. Exponential-phase bacteria were washed and resuspended (10^7^ CFU/ml) in sterile PBS containing 1 μM SYTOX Green (Molecular Probes). After 20 min of incubation in the dark, bacterial suspensions were mixed in PCR microplate wells with 50 μg/ml CRAMP or PBS (negative control) for a total volume of 100 μl. The mixtures were immediately placed at 37°C in a real-time PCR detection system (iQ5, Bio-Rad Laboratories) and fluorescence emission at 530 nm was recorded every minute following excitation at 488 nm.

### Binding of AMP to purified cell walls

One-hundred micrograms of purified cell wall were resuspended in 50 μl of 5 μg/ml CRAMP or PBS (negative control) and gently shaken for 5 min. Samples were centrifuged (16,000 × g, 1 min), washed in PBS and in TM buffer (10 mM Tris-HCl, 10 mM MgCl_2_, pH 7.4) before overnight incubation at 37°C with mutanolysin (400 U/ml) in TM buffer (50 μl). Supernatants were resolved by tricine-SDS-PAGE in a 16% gel, transferred onto nitrocellulose membrane and blotted with rabbit anti-CRAMP (1:1000) or mouse anti-InlA (L7.7; 1:1000), followed by HRP-conjugated goat anti-rabbit or anti-mouse IgG (1:2000, P.A.R.I.S). Immunolabeled bands were visualized using SuperSignal West Dura Extended Duration Substrate (Pierce) and digitally acquired in a ChemiDoc XRS+ system (Bio-Rad Laboratories).

### Immunoelectron microscopy

Exponential-phase bacteria treated with 50 μg/ml CRAMP for 15 min at 37°C were fixed for 1 h at room temperature (4% paraformaldehyde, 2.5% glutaraldehyde, 0.1 M sodium cacodylate, pH 7.2), stained with 1% osmium tetroxide for 2 h and resuspended in 30% BSA (high-purity grade). Bacterial pellets obtained after centrifugation in microhematocrit tubes were fixed overnight in 1% glutaraldehyde, dehydrated in increasing ethanol concentrations, and embedded in Epon 812. Ultrathin sections (40–50 nm) were placed on 400-mesh Formvar-coated copper grids and treated with 4% sodium metaperiodate and 1% periodic acid (10 min each) for antigen retrieval. For immunogold labeling of CRAMP, sections were blocked for 10 min with 1% BSA and incubated overnight (4°C) with rabbit anti-CRAMP (1:100 in 1% BSA). After extensive washing, sections were labeled with 10-nm gold complex-conjugated anti-rabbit IgG (1:200 in 1% BSA) for 2 h, washed and contrasted with 4% uranyl acetate and 1% lead citrate. Images were acquired in a Jeol JEM-1400 transmission electron microscope equipped with a Gatan Orius SC1000 CCD camera and analyzed using ImageJ software.

### Animal infections

Virulence studies were done in mouse models of the following strains: wild type BALB/c and 129/SvJ (Charles River Laboratories); and CRAMP-deficient (*cramp* ^-/-^) 129/SvJ, which was bred in our facilities from a breeding pair provided by Dr. Richard L. Gallo (University of California, USA) [49]. Infections were performed in six-to-eight week-old specific-pathogen-free females as described [76]. Briefly, for oral infections, 12-h starved animals were inoculated by gavage with 10^9^ CFU in PBS containing 150 mg/ml CaCO_3_, while intravenous infections were performed through the tail vein with 10^4^ CFU in PBS. In both cases, the infection was carried out for 72 h, at which point the animals were euthanatized by general anesthesia. The spleen and liver were aseptically collected, homogenized in sterile PBS, and serial dilutions of the organ homogenates plated in BHI agar. For analysis of *Lm* fecal carriage, total feces produced by each infected animal (n = 5 per strain) up to a given time-point were collected, homogenized in PBS and serial dilutions plated in *Listeria* selective media (Oxoid) for bacterial enumeration. Mice were maintained at the IBMC animal facilities, in high efficiency particulate air (HEPA) filter-bearing cages under 12 h light cycles, and were given sterile chow and autoclaved water *ad libitum*.

### Ethics statement

All the animal procedures were in agreement with the guidelines of the European Commission for the handling of laboratory animals (directive 2010/63/EU), with the Portuguese legislation for the use of animals for scientific purposes (Decreto-Lei 113/2013), and were approved by the IBMC Animal Ethics Committee, as well as by the Direcção Geral de Veterinária, the Portuguese authority for animal protection, under license PTDC/SAU-MIC/111581/2009.

### Statistical analyses

Statistical analyses were performed with Prism 6 (GraphPad Software). Unpaired two-tailed Student’s *t*-test was used to compare the means of two groups; one-way ANOVA was used with Tukey’s post-hoc test for pairwise comparison of means from more than two groups, or with Dunnett’s post-hoc test for comparison of means relative to the mean of a control group. Mean differences were considered statistically non-significant (ns) when *p* value was above 0.05. For statistically significant differences: *, *p*≤0.05; **, *p*≤0.01; ***, *p*≤0.001.

## Supporting Information

S1 Text Supporting figures and tables. **S1 Fig Proteins involved in *Lm* WTA l-rhamnosylation.** (**A**) Schematic diagram of the l-rhamnose biosynthesis pathway (adapted from [31, 35]). Each of the RmlACBD proteins catalyzes one of the four reaction steps that convert glucose-1-phosphate into nucleotide-linked l-rhamnose. dTTP, thymidine triphosphate; PP_i_, pyrophosphate; NADP, nicotinamide adenine dinucleotide phosphate. (**B**) Alignment of the amino acid sequences of *B*. *subtilis* 168 GgaB (GenBank: AAA73513.1) and *Lm* RmlT (GenBank: NP_464605.1). Boxed sequences correspond to the GT-A glycosyltransferase fold domain, as predicted by the NCBI Conserved Domain Search. The GT-A family signature DxD motif is highlighted in dark gray. The numbers indicate the position of the last amino acid in each line. Protein sequence alignments were obtained with ClustalW2 and edited with UCSF Chimera. **S2 Fig Genetic characterization of *Lm* strains used in this study.** (**A**) Genotypes and gene expression of the constructed *Lm* strains were confirmed by PCR and RT-PCR. (**B**) Comparison of the *rmlACBD* transcription levels in Δ*rmlT* versus wild type *Lm* strains by quantitative real-time PCR. Data represent the mean±SD of three independent analyses. *, *p*≤0.05. **S3 Fig HPLC analyses of the cell wall sugar and muropeptide composition from *Lm* strains.** (**A**) HPAEC-PAD analysis of the sugar composition of cell wall purified from *Lm* strains. Samples were hydrolyzed in 3 M HCl (2 h, 95°C), diluted with water and lyophilized before injection into the HPLC equipment. Standards for ribitol (Rib), l-rhamnose (Rha), glucosamine (GlcN), and muramic acid (Mur) were eluted under identical conditions to allow peak identification. (**B**) Reverse-phase HPLC analysis of the muropeptide composition from different *Lm* strains, following overnight digestion of purified peptidoglycan samples with mutanolysin and reduction with NaBH_4_. Muropeptide species (monomeric, dimeric, trimeric, etc.) were eluted with a 5–30% methanol gradient and detected by UV absorption at 206 nm. **S4 Fig Dose-dependent survival response of *Lm* strains to different AMPs.** Quantification of viable bacteria after treatment of mid-exponential-phase *Lm* strains (2 h, 37°C) with increasing concentrations of gallidermin, CRAMP or LL-37. The average replicate values from AMP-treated samples were expressed as percentage of surviving bacteria relative to the values of the respective untreated control samples (set at 100). Data represent mean±SD of three independent experiments. Asterisks indicate statistical significance between wild type and mutant strains (*, *p*≤0.05; ***, *p*≤0.001), while hashes indicate statistical significance between mutant and respective complemented strains (#, *p*≤0.05; ###, *p*≤0.001). **S5 Fig Zeta potential profile of wild type and WTA** **l** **-rhamnosylation mutant *Lm* strains. S6 Fig Determination of the *Lm* membrane potential magnitude by flow cytometry.** The membrane potential of untreated and sodium azide (1.5 mM)-treated suspensions of DiOC_2_(3)-stained wild type EGD-e suspensions was analyzed (see Materials and Methods) to determine the red/green fluorescence ratio values corresponding, respectively, to a basal (100%) and null (0%) membrane potential. **S7 Fig SYTOX Green uptake kinetics of *Lm* strains in response to CRAMP-mediated membrane permeabilization.** Exponential-phase bacteria were incubated (37°C) with PBS (white symbols) or 50 μg/ml CRAMP (black symbols), in the presence of 1 μM SYTOX Green, and the increase in green fluorescence emission was recorded over 115 min. **S8 Fig Growth of *Lm* strains in broth and inside eukaryotic host cells.** (**A**) Stationary-phase cultures were diluted 100-fold in BHI broth and incubated at 37°C in aerobic and shaking conditions. Optical density values at 600 nm (OD_600_) from each culture were measured every hour. (**B**) Intracellular multiplication in J774A.1 murine macrophages. Cells (2×10^5^/well) were infected (45 min) with *Lm*, treated with 20 μg/ml gentamicin (75 min) and lysed at 2, 5, 7 and 20 h post-infection for quantification of intracellular viable bacteria in BHI agar. **S1 Table. Homology between the RmlACBD proteins of *Lm* EGD-e and other strains and species. S2 Table. Primers.**
(PDF)
