# Supplementary material for: L-Rhamnosylation of Listeria monocytogenes Wall Teichoic Acids Promotes Resistance to Antimicrobial Peptides by Delaying Interaction with the Membrane
Source: PLoS Pathog. 2015 May 22;11(5):e1004919. doi: 10.1371/journal.ppat.1004919 (PMC4441387; doi:10.1371/journal.ppat.1004919)
Supplement: S1 Text — S1 Fig Proteins involved in Lm WTA l-rhamnosylation. (A) Schematic diagram of the l-rhamnose biosynthesis pathway (adapted from [31, 35]). Each of the RmlACBD proteins catalyzes one of the four reaction steps that convert glucose-1-phosphate into nucleotide-linked l-rhamnose. dTTP, thymidine triphosphate; PPi, pyrophosphate; NADP, nicotinamide adenine dinucleotide phosphate. (B) Alignment of the amino acid sequences of B. subtilis 168 GgaB (GenBank: AAA73513.1) and Lm RmlT (GenBank: NP_464605.1). Boxed sequences correspond to the GT-A glycosyltransferase fold domain, as predicted by the NCBI Conserved Domain Search. The GT-A family signature DxD motif is highlighted in dark gray. The numbers indicate the position of the last amino acid in each line. Protein sequence alignments were obtained with ClustalW2 and edited with UCSF Chimera. S2 Fig Genetic characterization of Lm strains used in this study. (A) Genotypes and gene expression of the constructed Lm strains were confirmed by PCR and RT-PCR. (B) Comparison of the rmlACBD transcription levels in ΔrmlT versus wild type Lm strains by quantitative real-time PCR. Data represent the mean±SD of three independent analyses. *, p≤0.05. S3 Fig HPLC analyses of the cell wall sugar and muropeptide composition from Lm strains. (A) HPAEC-PAD analysis of the sugar composition of cell wall purified from Lm strains. Samples were hydrolyzed in 3 M HCl (2 h, 95°C), diluted with water and lyophilized before injection into the HPLC equipment. Standards for ribitol (Rib), l-rhamnose (Rha), glucosamine (GlcN), and muramic acid (Mur) were eluted under identical conditions to allow peak identification. (B) Reverse-phase HPLC analysis of the muropeptide composition from different Lm strains, following overnight digestion of purified peptidoglycan samples with mutanolysin and reduction with NaBH4. Muropeptide species (monomeric, dimeric, trimeric, etc.) were eluted with a 5–30% methanol gradient and detected by UV absorption at 206 nm. S4 [file ppat.1004919.s001.pdf]

A

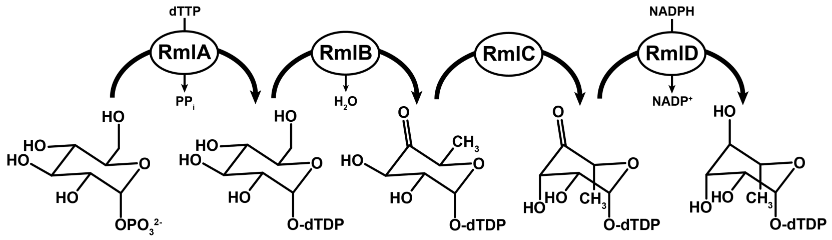

glucose 1-phosphate | dTDP-glucose | dTDP-4-keto-6-deoxy-glucose | dTDP-L-lyxo-6-deoxy-4-hexulose | dTDP-L-rhamnose

B

```
Bsu_GgaB  -----------MNEKSFNYDFSVIMPIYNVELY-LTEAIESIINQTIGFE 38
Lmo_RmlT  MRNLKDRVLNSLKGNKKDIKISVVVPTYNTELEGLKNLMASIDKQTMNPD 50

Bsu_GgaB  NIQLILVNDDSPDKSEIICKEYAQKYPNNIVYAKKQNGGVSSARNYGLKY 88
Lmo_RmlT  EYELVFVDDGSTTDTYERLQEFAETRPNMTVKQIENSGWGSRPRNIATKM 100

Bsu_GgaB  AEGRYIQFLDPDDLVSEGTFENVLNFFDEHKNEIDIVAIPIFFAEGRTGE 138
Lmo_RmlT  AKGEYILYLDHDDTVFPETFERVYNFGKENN--LDVVSGKEVRTNGWSWG 148

Bsu_GgaB  HNLNNKFSSTRILDVEKEPHHILTHCCSTFIKKDALKN--IRFDENCKIG 186
Lmo_RmlT  ---WKQFSENNPHAEEMGIECLLPMTPHKFYKREFLLENDITFDDGARVL 195

Bsu_GgaB  -EDAKLVNLIISQKKKYGLVKEAKYHYRVREDGSSAMQTAKANKNWFNHS 235
Lmo_RmlT  WEDVYFNSKAFIHGAKVGILADYPTYYWIATGANNSSSFGRDPHEKWNQI 245

Bsu_GgaB  --LITFSKNLIDIIKNHEQKIPLFLQYMVMHDLKWKLLIKDISETPLDEN 283
Lmo_RmlT  NKLFNFFKDNIKEQRDLDFMLTHWYRSRVLGILGQWLLKNNNERIDIEFN 295

Bsu_GgaB  EYSEFLTLIREVLSYIDDDVIIETKSVSHFYLYHALKIKHGENYSRYVYE 333
Lmo_RmlT  -------------------------------YAKKLAEELIPAYISENLD 314

Bsu_GgaB  RETEQDYYLYREG--KIVSKLSDQTLTIEILEENEDSIHIEGFWSSLFNS 381
Lmo_RmlT  KNNQVKDYLLRQGDLDSLKKLAQIDAGITALSYVEDAYFKE---DKLFFK 361

Bsu_GgaB  KGFKFYAKIGETKIKAKNIKRQHNDYISLGEVIKKYPGFSIDIPKGHLAD 431
Lmo_RmlT  TSTKMTYEDKEDFFIEKTADRMER--ILPEEIKSKLPKEFFDYSD-DLAE 408

Bsu_GgaB  NHHIEFFITKGKKRKLTKLRFFKYSGLSNDLYNTYVAKKDYIFYYNYKKL 481
Lmo_RmlT  FTYEPSIKGRNSRATWKIDGSTSNVEVVNKKANLYKIEGEMSFSVQINDY 458

Bsu_GgaB  MFKKNNFKNRFIKEFRFLKSLHKSGEKSKKRKSAIKKALMARMVHHVFTI 531
Lmo_RmlT  ILDAADKKQPWDIATRFTGLGYTSHRALTIGKILIKTALIN---NKTMIV 505

Bsu_GgaB  FNRKPVWLFIDRQDKADDNAEHLFKYAINKNDGVKKYFIIKKDSKDYDRI 581
Lmo_RmlT  YKNASGLISLDVGSSVRS---------IVEDSGVKREQILIDKTSG---- 542

Bsu_GgaB  KKYGKVIPYRSFRHKILTLSSSKVISTHADIWVVNPFFNMEIYFRDLFNF 631
Lmo_RmlT  -------------------------------KVTIPLNEIHVFGESLIEG 561

Bsu_GgaB  EFIFLQHGITMADHSEWLNKYNKNIKLLVTSAKPEYRSIVKGNYNYKKEN 681
Lmo_RmlT  NAELKPVGISDADP------INVKAKLIGEANKARVEVLLG-------DE 598

Bsu_GgaB  ILLGGFPRYDNLKKSEGEKQLLIMPTWRKDIVLPKDQAKGVRPYNPKFKD 731
Lmo_RmlT  KLSGEYHLVTNIQGKKDKQQIKITL------------------------- 623

Bsu_GgaB  SEYFSRYNALINDERLIEFAKKNNYKITFFPHPDIQQQIVDFEKHDYVEF 781
Lmo_RmlT  -------------------------------------------------- 623

Bsu_GgaB  ADYNSSYQMLFNSSNIMITDFSSVAFDFAYEKKPVIYYQYEKSYHFKLDY 831
Lmo_RmlT  -------------------------------------------------- 623

Bsu_GgaB  YDYKKMGFGDVLENHNSLVDKVIYYMKNNSRMEDKYRKRVDNFFAYTDKN 881
Lmo_RmlT  -------------------------------------------------- 623

Bsu_GgaB  NRNRIYNAILELDNNKVAK 900
Lmo_RmlT  ------------------- 623
```

Figure S1

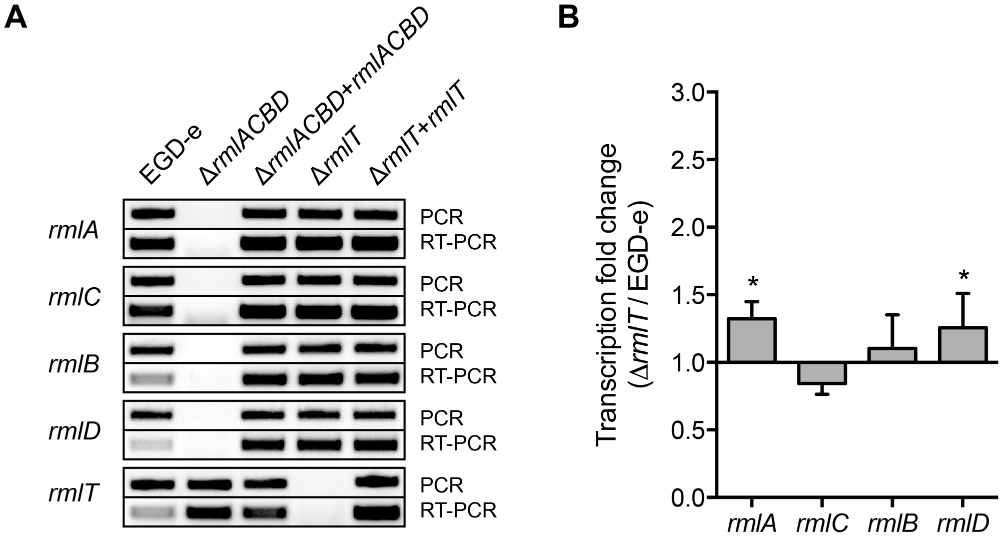
A
EGD-e
ΔrmlACBD
ΔrmlACBD+rmlACBD
ΔrmlT
ΔrmlT+rmlT
rmlA
rmlC
rmlB
rmlD
rmlT
PCR
RT-PCR
B
Transcription fold change (ΔrmlT / EGD-e)
3.0
2.5
2.0
1.5
1.0
0.5
0.0
*
*
rmlA
rmlC
rmlB
rmlD

Figure S2

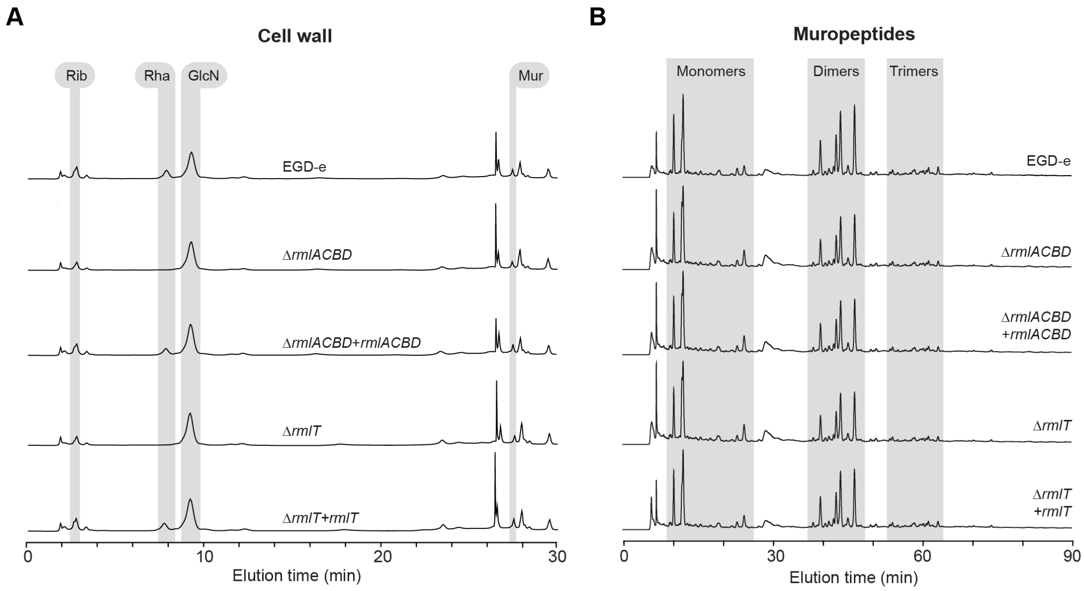

A
Cell wall
Rib
Rha
GlcN
Mur
EGD-e
ΔrmlACBD
ΔrmlACBD+rmlACBD
ΔrmlT
ΔrmlT+rmlT
0
10
20
30
Elution time (min)
B
Muropeptides
Monomers
Dimers
Trimers
EGD-e
ΔrmlACBD
ΔrmlACBD
+rmlACBD
ΔrmlT
ΔrmlT
+rmlT
0
30
60
90
Elution time (min)

Figure S3

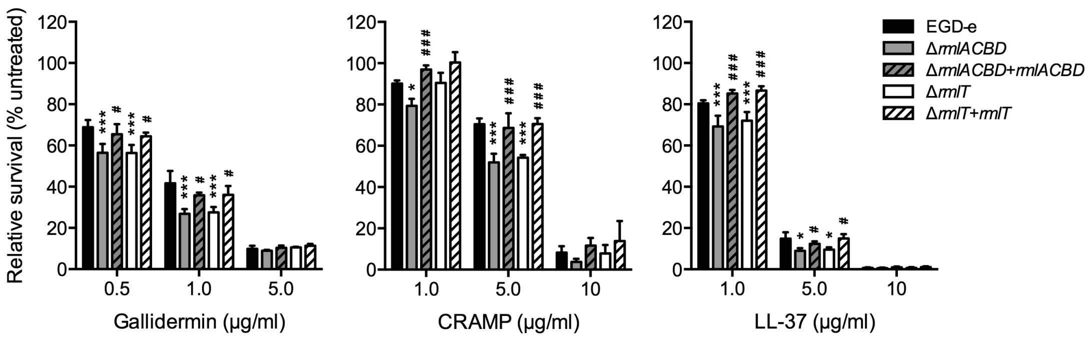

Relative survival (% untreated)
Gallidermin (µg/ml)
CRAMP (µg/ml)
LL-37 (µg/ml)
EGD-e
Δ*rmlACBD*
Δ*rmlACBD*+*rmlACBD*
Δ*rmlT*
Δ*rmlT*+*rmlT*

Figure S4

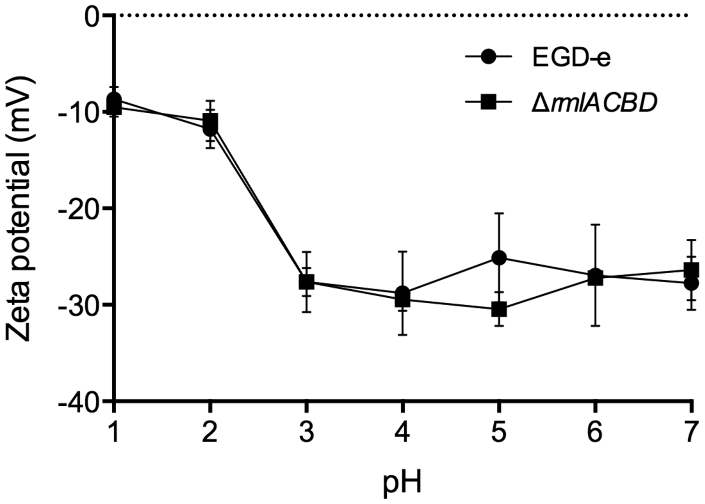

Figure S5

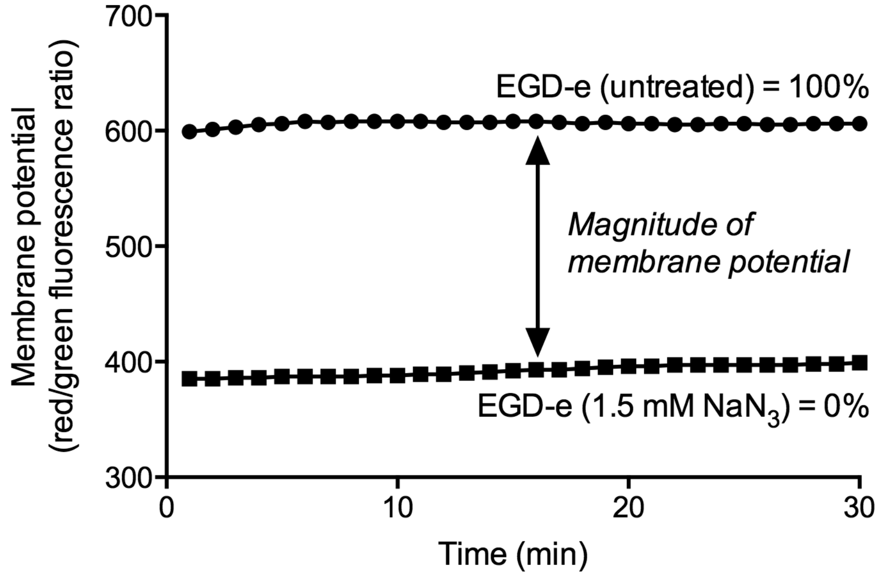
700
600
500
400
300
0
10
20
30
Membrane potential
(red/green fluorescence ratio)
Time (min)
EGD-e (untreated) = 100%
Magnitude of
membrane potential
EGD-e (1.5 mM NaN3) = 0%

Figure S6

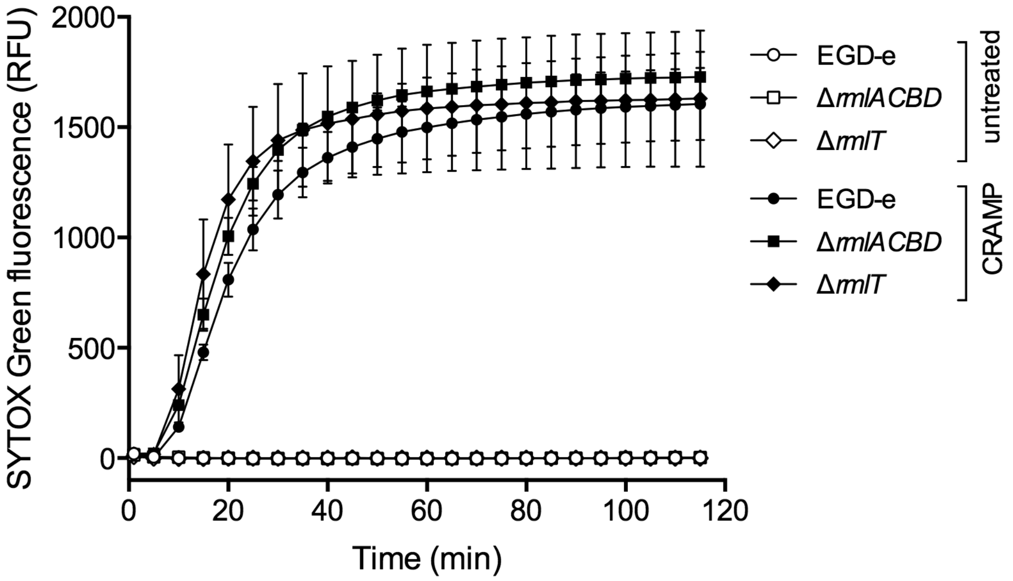
SYTOX Green fluorescence (RFU)
2000
1500
1000
500
0
0
20
40
60
80
100
120
Time (min)
EGD-e
ΔrmlACBD
ΔrmlT
untreated
EGD-e
ΔrmlACBD
ΔrmlT
CRAMP

Figure S7

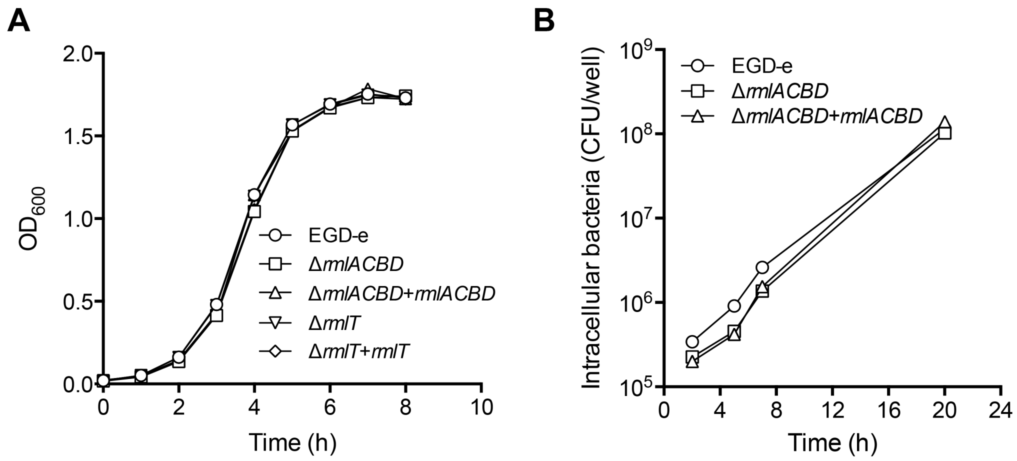
A
B
2.0
1.5
1.0
0.5
0.0
OD600
0
2
4
6
8
10
Time (h)
EGD-e
ΔrmlACBD
ΔrmlACBD+rmlACBD
ΔrmlT
ΔrmlT+rmlT
Intracellular bacteria (CFU/well)
10^9
10^8
10^7
10^6
10^5
0
4
8
12
16
20
24
Time (h)
EGD-e
ΔrmlACBD
ΔrmlACBD+rmlACBD

Figure S8

Table S1. Homology between the RmlACBD proteins of *Lm*EGD-e and other strains and species[a]

Species[b] / Strain	Serovar	RmlA	RmlB	RmlC	RmlD
Lmo 10403S	1/2a	100	100	100	100
Lmo SLCC2755	1/2b	100	99	99.5	99.3
Lmo SLCC2372	1/2c	100	100	100	100
Lmo SLCC2479	3c	100	100	100	100
Lmo SLCC2482	7	100	98.7	99.5	99.3
Lse SLCC3954	1/2b	97.2	95.1	98.4	92.0
Smu UA159		74.6	45.7	28.6	51.6
Mtu H37Rv		58.3	46.7	33.5	34.3
Sen LT2		68.4	51.8	46.4	34.8
Sfl 2457T		70.8	51.5	48.0	35.9
Pae PAO1		69.1	52.4	47.2	32.2

[a]Values in percentage of amino acid identity determined by protein-protein BLAST analysis

[b]*Lmo*, *Listeria monocytogenes*; *Lse*, *Listeria seeligeri*; *Smu*, *Streptococcus mutans*; *Mtu*, *Mycobacterium tuberculosis*; *Sen*, *Salmonella enterica* serovar Typhimurium; *Sfl*, *Shigella flexneri*; *Pae*, *Pseudomonas aeruginosa*

Table S2. Primers

#	Name	Sequence (5' to 3')[a]
Construction of plasmids and screening of clones		
1	*rmlA*-A	TACGTCGACTGCTCAAATCGATGCTGG
2	*rmlA*-B	CGACGCGTCATTCTTTTCTCTCC
3	*rmlD*-C	ATACGCGTTTGGCAAGATGCTTTAGTTCG
4	*rmlD*-D	ATTAGATCTTAGTGGTCTCCACCAAGC
5	*rmlA*-F	GGCTACCACGTGAATGATCC
6	*rmlA*-R	AACTCACCACGTTCAGATGG
7	*rmlB*-F	GCAGCAGAATCTCATGTAGACC
8	*rmlB*-R	CCAGTTTCTCCAAGTGAACC
9	*rmlC*-F	ACATACGGTGAGTGGGAAGG
10	*rmlC*-R	AATCCGGATCATCGTAGGC
11	*rmlD*-F	TGGGAAGTAAACGTGGATGG
12	*rmlD*-R	CCAAACACCCATGAAGTACG
13	*rmlA*-G	ATACTATGCGGCCGCTTCATGTGTTTGGTGAAAGC
14	*rmlD*-H	GCGGTCGACACAATTATACGAATGCATCG
15	*rmlT*-A	ATAGTCGACCCTAAAGTTAATGGCAAAGCTCCTGC
16	*rmlT*-B	CGAATTCCATTATATCCTCCTAAAATAGATTAACAG
17	*rmlT*-C	CGAATTCTAAGAATGGAGAGAAAAGAATGAAAGG
18	*rmlT*-D	ACTAGATCTCAATTTCCATTAGTACGCCTCACTC
19	*rmlT*-F	TATTGCCACACGCTTTACCG
20	*rmlT*-R	CTTCCACGATTGAACGAACG
21	*rmlT*-G	TATCTGCAGGAGGGAAAACGTTAGGTAGC
22	*rmlT*-H	GCGGTCGACCTAGTTCCACTTCCTCCTGC
23	PL95	ACATAATCAGTCCAAAGTAGATGC
24	PL102	TATCAGACCTAACCCAAACCTTCC
Quantitative real-time PCR		
25	qPCR-*rmlA*-F	TTCTTGAAGCGTCTACCT
26	qPCR-*rmlA*-R	GCAGCCTCATCAATATACC
27	qPCR-*rmlB*-F	GTAGACCGTAGTATTATCAATCC
28	qPCR-*rmlB*-R	TCTCCAAGTGAACCATACA
29	qPCR-*rmlC*-F	TATTCAAGATAACCACTC
30	qPCR-*rmlC*-R	TCAACAACTACATCATAA
31	qPCR-*rmlD*-F	AGATTCTGTAGATATTGTGGAT
32	qPCR-*rmlD*-R	CATCTTCTGCTGCTTCTA
32	qPCR-16S-F	GCGTAGATATGTGGAGGAAC
33	qPCR-16S-R	CAGGCGGAGTGCTTAATG

[a] Restriction sites underlined
